# In Situ Deposition of Drug and Gene Nanoparticles on a Patterned Supramolecular Hydrogel to Construct a Directionally Osteochondral Plug

**DOI:** 10.1007/s40820-023-01228-w

**Published:** 2023-11-17

**Authors:** Jiawei Kang, Yaping Li, Yating Qin, Zhongming Huang, Yifan Wu, Long Sun, Cong Wang, Wei Wang, Gang Feng, Yiying Qi

**Affiliations:** 1 Department of Orthopaedic Surgery, The Second Affiliated Hospital, Zhejiang University School of Medicine, Hangzhou City, 310009 Zhejiang Province People’s Republic of China; 2 College of Chemical and Biological Engineering, Zhejiang University, Hangzhou, 310027 People’s Republic of China; 3 ZJU-Hangzhou Global Scientific and Technological Innovation Center, Hangzhou, 311215 Zhejiang People’s Republic of China; 4 The Affiliated Nanhua Hospital, Orthopedic Research Centre, Hengyang Medical School, University of South China, Hengyang, 421001 Hunan People’s Republic of China; 5 Department of Radiology, Jining No. 1 People’s Hospital, Jining Medical University, Jining, 272000 Shandong People’s Republic of China

**Keywords:** Osteochondral regeneration, Oriented hydrogel, Kartogenin, miRNA-26a

## Abstract

The integrated repair of bone and cartilage boasts advantages for osteochondral restoration such as a long-term repair effect and less deterioration compared to repairing cartilage alone. Constructing multifactorial, spatially oriented scaffolds to stimulate osteochondral regeneration, has immense significance. Herein, targeted drugs, namely kartogenin@polydopamine (KGN@PDA) nanoparticles for cartilage repair and miRNA@calcium phosphate (miRNA@CaP) NPs for bone regeneration, were in situ deposited on a patterned supramolecular-assembled 2-ureido-4 [lH]-pyrimidinone (UPy) modified gelation hydrogel film, facilitated by the dynamic and responsive coordination and complexation of metal ions and their ligands. This hydrogel film can be rolled into a cylindrical plug, mimicking the Haversian canal structure of natural bone. The resultant hydrogel demonstrates stable mechanical properties, a self-healing ability, a high capability for reactive oxygen species capture, and controlled release of KGN and miR-26a. In vitro, KGN@PDA and miRNA@CaP promote chondrogenic and osteogenic differentiation of mesenchymal stem cells via the JNK/RUNX1 and GSK-3β/β-catenin pathways, respectively. In vivo, the osteochondral plug exhibits optimal subchondral bone and cartilage regeneration, evidenced by a significant increase in glycosaminoglycan and collagen accumulation in specific zones, along with the successful integration of neocartilage with subchondral bone. This biomaterial delivery approach represents a significant toward improved osteochondral repair.

**Supplementary Information:**

The online version contains supplementary material available at 10.1007/s40820-023-01228-w.

## Highlights

- A multifactorial and oriented scaffolds was constructed to stimulate osteochondral regeneration.
- This is the first demonstration that both drug and gene nanoparticles were spatially deposited on a patterned film through metal-ligand interactions.
- For the first time, film-rolling approach was employed to construct osteochondral plug to mimick the Haversian canal structure of natural bone.

**Supplementary Information:**

The online version contains supplementary material available at 10.1007/s40820-023-01228-w.

## Graphical abstract

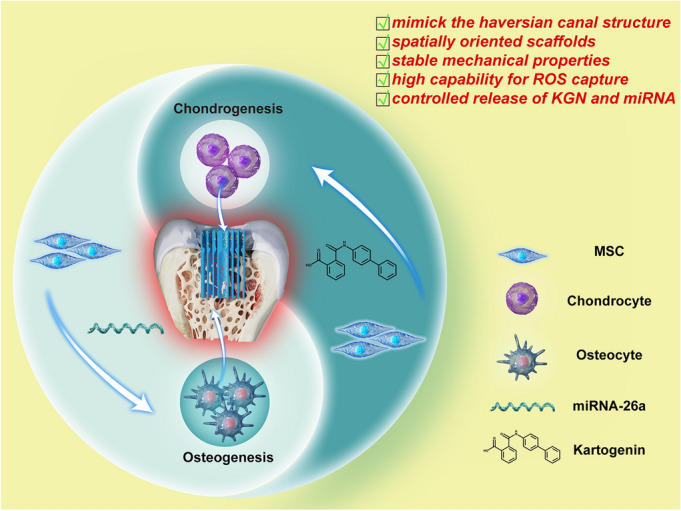

## Introduction

Osteochondral regeneration involving both bone and cartilage tissues is a pressing issue in clinical situations. The concurrent regeneration of bone and cartilage is challenging to achieve due to their disparate nature. The vertical integration of neocartilage with subchondral bone and the subsequent construction of the subchondral bone are crucial for mitigating tissue deterioration [1]. Many studies in the literature primarily focused on cartilage scaffolds for osteochondral tissue engineering, but often neglected the need for subchondral bone regeneration [2]. The development of engineered scaffolds that mimicing both cartilage and bone, incorporating distinct growth factors for their concurrent regeneration, remains a significant obstacle in osteochondral tissue engineering [3, 4].

The key to achieving high-quality repair lies on constructing an osteochondral scaffold with a biomimetic hierarchy of the native tissue, including both the articular cartilage and subchondral bone, and biological functionality. Numerous techniques have been developed to fabricate bilayer or multilayer osteochondral scaffolds, such as sequential layering of slurry or hydrogel solutions, 3D printing, electrospinning, microfluidic-based methods, magnetic field control, and buoyancy-driven approach [4]. An exemplar of this is an injectable and 3D-printable bilayered osteochondral hydrogel, developed based on a compositional gradient of methacrylated sodium alginate, gelatin methacryloyl, and β-tricalcium phosphate (β-TCP), along with the biochemical gradient of kartogenin (KGN) in the two well-integrated zones of chondral layer hydrogel and osseous layer hydrogel. This has been designed to promote osteochondral regeneration [5]. However, all these methods involve complex multi-step manufacturing processes that impeding their practical applications. Drawing inspiration from the Haversian canal structure of natural bone, a conventional film-rolling approach can be employed to fabricate a well-designed osteochondral scaffold.

Due to the distinct heterogeneity of cartilage and bone, different stimulating factors need to be spatially loaded into various parts of the scaffold and responsively released in a sustained manner for osteochondral repair. A number of researchers have discovered various approaches to load drug/gene nanoparticles in various types of scaffolds. A case in point, the dominantly used drug-eluting stents shows a promising potential by loading various drugs in the stents to regulate macrophage polarization, reduce local inflammation, and promote tissue repair [6, 7]. Due to the dynamic and reversible property, physical affinity is a much more powerful approach to load drugs within scaffolds than that achieved by physical adsorption and covalent crosslinking [8]. The gene-activated surface coating is recently explored as a strategy to design smart biomaterials for tissue engineering. Husteden et al. incorporated DNA/lipid-nanoparticles (lipoplexes) into collagen I/chondroitin sulfate polyelectrolyte multilayers, fabricated through a layer-by-layer assembly on a bone scaffold to effectively promote bone regeneration [9]. Metal-ligand interaction is one of the most prevailing strategies to integrate the stimulated factors in scaffolds. Yang et al. developed a stepwise metal-catechol surface chemistry strategy, leading to a stable nitric oxide (NO) generating rate and a high heparin conjugation on the cardiovascular stents [10]. We believe that by optimizing metal ions and their ligands, different types of drugs/genes can be spatially tethered on a patterned film through an in situ metal-ion-assisted deposit approach.

**Fig. 1:**
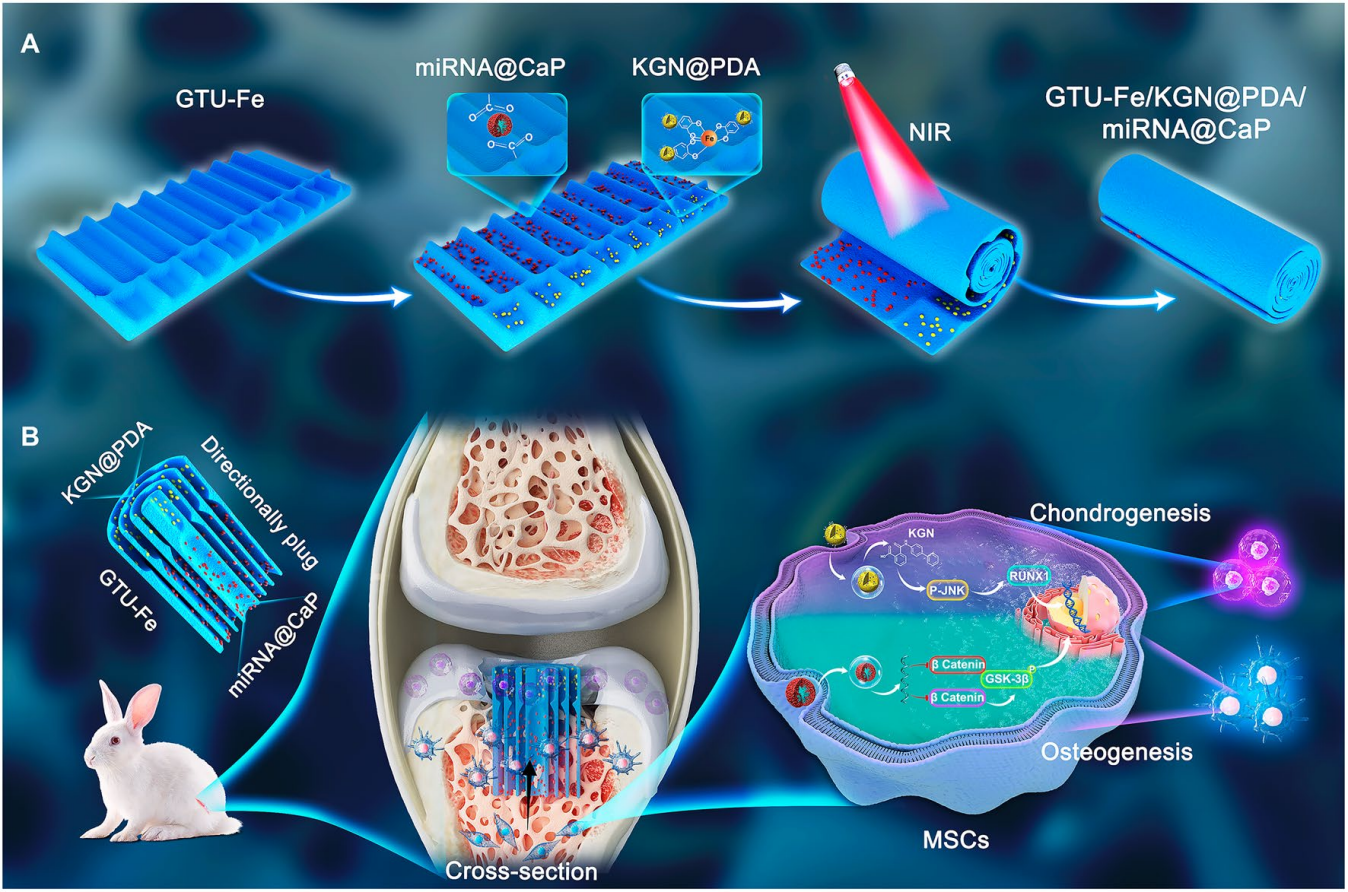
Schematic illustrating the design of oriented GTU-Fe/KGN@PDA/miRNA@CaP hydrogel for repairing osteochondral defects. **A** The fabrication process of oriented GTU-Fe/KGN@PDA/miRNA@CaP hydrogel film; **B** Schematic construct to repair osteochondral defects

Nanoparticles (NPs) refer to submicron particles with sizes ranging from 1 to 100 nm. Nanoparticles as carriers can be incorporated into the surface or matrix of drugs to protect them from enzymatic degradation, improve their permeability to the cartilage matrix, and regulate drug pharmacokinetics, which is beneficial for balancing the efficacy and toxicity of therapeutic compounds [11]. In this work, as shown in Fig. 1, we adopted an in situ deposition strategy to achieve a spatial distribution of drug NPs and gene miRNA@CaP NPs on a patterned hydrogel film. Initially, a patterned supramolecular-assembled 2-ureido-4 [lH]-pyrimidinone (UPy) modified gelation (GTU) hydrogel film was fabricated by enhancing solidification with Fe^3+^. KGN, a non-protein small chondrogenesis molecule [12–14], was encapsulated by polydopamine (PDA) to form KGN@PDA NPs, which were anchored in the cartilage layer to promote cartilage repair. KGN has been associated with effective results in tissue repair and regeneration in various studies, including cartilage regeneration [15], cartilage protection [16], tendon-bone healing [17], wound healing [18], and limb development [19]. Simultaneously, miRNA-26a (miR-26a), a promoter of osteogenic differentiation of mesenchymal stem cells (MSCs) [20, 21], was co-deposited with calcium phosphate (CaP) to create miRNA@CaP NPs, which were immobilized in the bone layer to facilitate bone regeneration. miR-26a plays a crucial role in other biological functions, such as regulating diabetes mellitus-related bone metabolism [22], reducing inflammatory responses [23] and promoting angiogenesis [24, 25]. The complexation effect of the catechol group and iron ion allows the KGN@PDA NPs to dynamically anchor onto the surface of the GTU-Fe hydrogel, while the coordination between the calcium ion and the carboxyl group of gelatin facilitates the immobilization of miRNA@CaP on the GTU-Fe hydrogel. Subsequently, the resulting hydrogel film was rolled into a cylindrical plug to mimic the Haversian canal structure of natural bone. This directionally bionic hydrogel scaffold is capable of controlled in situ release of KGN in the upper layer for cartilage repair and miR-26a in the bottom layer for subchondral regeneration. This novel design, combining dual-targeting bioactive factors with a longitudinally oriented structure to mimic osteochondral tissue, holds great clinical potential for effective cartilage and subchondral bone regeneration.

## Experimental Section

### Preparation of KGN@PDA and miRNA@CaP NPs

KGN@PDA NPs were fabricated using a typical reprecipitation method, as described in our previous reports [26]. Specifically, 100 μL of a 5 mg mL^−1^ KGN solution in dimethyl sulfoxide was added dropwise to 5 mL of water, forming a KGN NP aqueous dispersion. Subsequently, dopamine (6 mg) was dissolved in 5 mL of buffer solution (pH 8.5). An equal volume of the KGN NP aqueous dispersion was then added, and the self-polymerization of dopamine was allowed to proceed under stirring for 24 h. The resulting suspension was centrifuged at 12,000 rpm and rinsed three times with 50 mL of deionized water to obtain the KGN@PDA NPs.

miRNA@CaP NPs were fabricated as previously reported [27]. Briefly, miR-26a was added to a 7 mM CaCl_2_ solution, which was then mixed with an equal volume of a 4.2 mM Na_2_HPO_4_ solution to produce miRNA@CaP NPs, with Na_3_Cit used as a stabilizer.

### Preparation of GTU Hydrogel

Gelatin-UPy was used as the matrix to in situ load the resulting NPs. Gelatin-UPy was synthesized through a typical two-step method as per previous reports [28]. The GTU-Fe hydrogel was prepared by mixing a GTU solution (20 wt%) with a FeCl_3_ solution (12 mM). This mixture was then homogenized using a vortex at 3000 rpm for 10 s. The resulting hydrogel precursor solution was poured into a customized mold (Fig. S1) and left for 24 h, then removed from the mold to acquire the patterned hydrogel film. Gelatin-Fe hydrogel was also prepared following the same procedure, except that gelatin was used to replace gelatin-UPy, which is referred to as G-Fe.

### Characterization of Samples

The resulting NPs were characterized using dynamic light scattering (DLS; Nano ZS; Malvern Instruments, Malvern, UK), a high-resolution transmission electron microscope (HRTEM; JEM-2100F; JEOL, Tokyo, Japan), a field-emission scanning electron microscope (SEM; Gemini SEM 300; Zeiss, Jena, Germany), and X-ray diffraction (XRD; D8-Advance; Bruker, Fällanden, Switzerland). The mechanical properties of the hydrogels were characterized by a rheometer (DHR-2; TA, USA) and a computerized electronic universal testing machine (UTM2102; SUNS, Shenzhen, China).

## Results and Discussion

### Properties of KGN@PDA and miRNA@CaP NPs

**Fig. 2:**
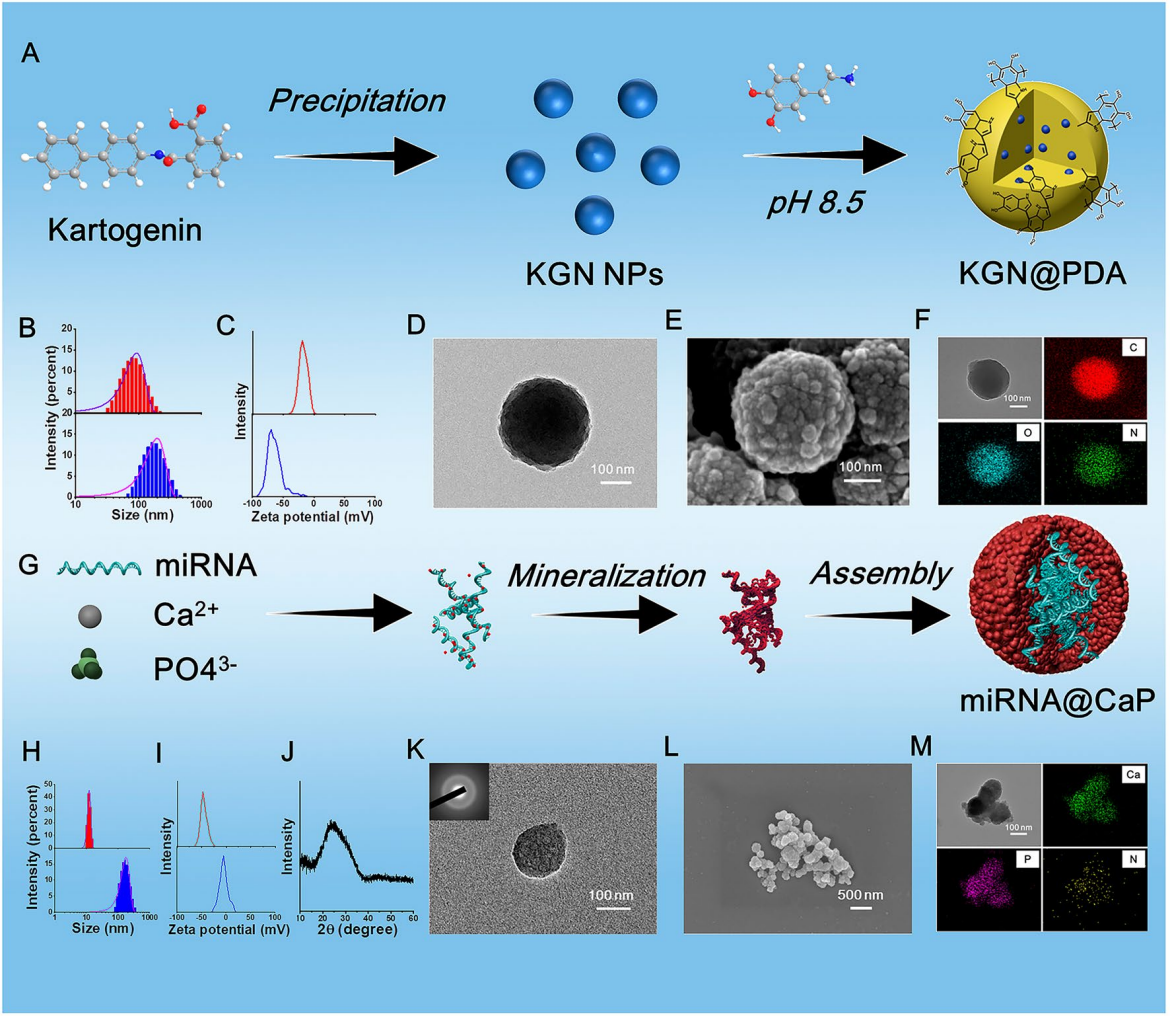
Preparation and characterizations of KGN@PDA and miRNA@CaP NPs. **A** Schematic diagram of the preparation of KGN@PDA. **B, C** Size distribution and zeta potential of KGN and KGN@PDA. **D, E** Morphology of KGN@PDA observed by TEM and SEM. **F** Elemental distribution of KGN@PDA. **G** Schematic diagram of the preparation of miRNA@CaP. **H, I** Size distribution and zeta potential of miRNA@CaP. **J** XRD pattern of miRNA@CaP. **K** TEM images and SAED pattern of miRNA@CaP. **L** Morphology of miRNA@CaP observed by SEM. **M** Elemental distribution of miRNA@CaP

As shown in Fig. 2A, the synthesis of KGN@PDA involves two stages. First, the KGN core is prepared via a reprecipitation method. Specifically, a solution of KGN/DMSO (1 mL) is gradually added to deionized water (100 mL) while stirring vigorously. Due to the abrupt change in solvent properties, KGN molecules can aggregate via hydrophobic effects and precipitate to form KGN NPs. Subsequently, the PDA shell is applied to coat the KGN NPs. As shown in Fig. 2B, the KGN NPs have an average size of about 95.4 nm, while KGN@PDA NPs have an average size of approximately 288.4 nm, suggesting that multiple KGN particles might form a single KGN@PDA particle. The zeta potential of KGN is - 22.0 mV, whereas the potential of KGN@PDA is -63.3 mV. The HRTEM image clearly shows that KGN@PDA NPs have a spherical morphology with a diameter of around 200-300 nm. Irregular sheet-like structures can be seen assembled together on the KGN@PDA surface when observed by SEM, these structures consist of spherical KGN coated by PDA. As illustrated in Fig. 2F, the elements C, O, and N in KGN@PDA are evenly distributed. It has been found that catechol groups on PDA can further complex with iron ions [26]. Therefore, KGN@PDA can be complexed with GTU-Fe hydrogel for in situ deposition on the hydrogel in subsequent studies.

As shown in Fig. 2G, miRNA@CaP was fabricated using a typical calcium phosphate co-precipitation technique. miR-26a has an average hydrodynamic diameter of 12.3 nm and a zeta potential of -42.2 mV, while miRNA@CaP has an average hydrodynamic diameter of 170.7 nm and a zeta potential of -1.64 mV (Fig. 2H, I). The XRD pattern of the miRNA@CaP nanocomposite exhibits a broad band at approximately 20=25° (Fig. 2J), suggesting a lack of long-range periodicity and excluding the presence of other CaP crystalline phases. The nanostructure and crystallization pattern of miRNA@CaP were characterized by HRTEM in combination with selected area electron diffraction (SAED) (Fig. 2K). The SAED patterns do not show the characteristic dot or ring patterns of crystal structures, indicating the amorphous nature of miRNA@CaP. HRTEM, and SEM images clearly show that miRNA@CaP NPs have an irregular spherical shape. As demonstrated in Fig. 2M, miR-26a-derived nitrogen is mainly distributed in the center of miRNA@CaP complexes, whereas calcium and phosphorus are located along the periphery of NPs in the elemental mapping. Concurrently, the interaction between calcium ions and the hydroxyl and carboxyl groups of the GTU-Fe hydrogel enables miRNA@CaP to be deposited in situ on the hydrogel pattern, leading to a mineralized hydrogel in subsequent steps.

### Properties of GTU-Fe/KGN@PDA/miRNA@CaP Hydrogel

**Fig. 3:**
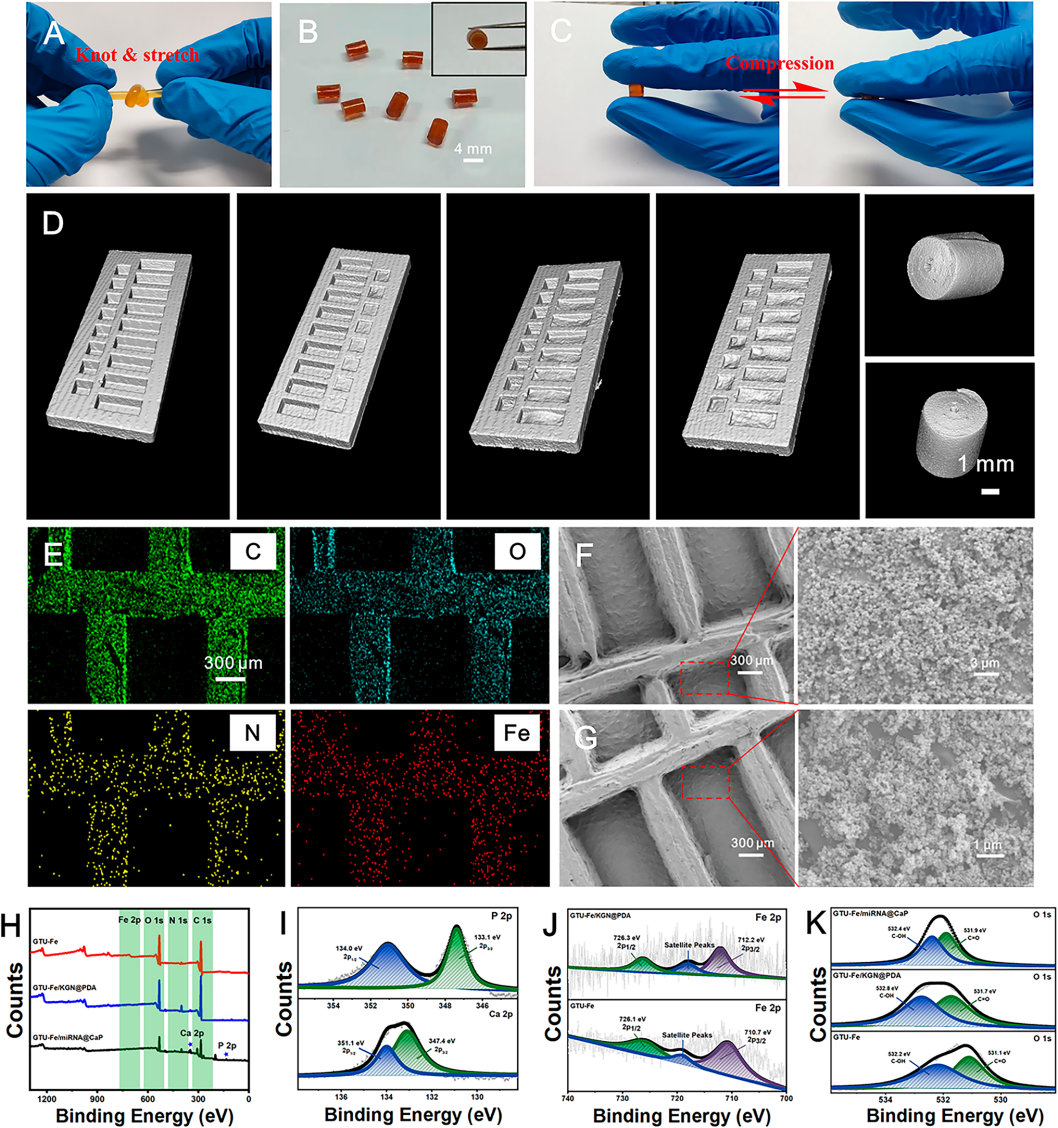
Characterizations of GTU-Fe/KGN@PDA/miRNA@CaP. **A, B** Photograph of GTU-Fe and GTU-Fe/KGN@PDA/miRNA@CaP hydrogel scaffold. **C** GTU-Fe/KGN@PDA/miRNA@CaP hydrogel scaffold recovered completely under compression. **D** Micro-CT scan images of the hydrogels. **F, G** Morphology of GTU-Fe/KGN@PDA/miRNA@CaP hydrogel scaffold. **H** Total survey scans of XPS spectra. **I** Ca 2p and P 2p spectrum of GTU-Fe/miRNA@CaP. **J** Fe 2p spectra of GTU-Fe hydrogel and GTU-Fe/KGN@PDA. **K** O 1s spectra of GTU-Fe hydrogel, GTU-Fe/KGN@PDA and GTU-Fe/miRNA@CaP

As demonstrated in Fig. S2-S4, the UPy-NCO, GTU, and supramolecular GTU-Fe hydrogel were synthesized, and the chemical structure was confirmed by ^1^H-NMR, UV-vis and FTIR. The resulted GTU hydrogel shows a porous morphology as the conventional gelatin scaffolds. As shown in Fig. 3, the physical double-network GTU-Fe hydrogel with robust viscoelasticity, rapid self-healing, and near-infrared (NIR) stimulus responsiveness was synthesized to serve as the matrix for loading drug/gene NPs. The graft rate can be effectively controlled by the feeding ratio of gelatin and UPy-NCO, with a moderate graft rate of 6.12% chosen for further studies. The resulting GTU-Fe hydrogel can withstand knotting and stretching (Fig. 3A) due to the stable physical double-network crosslinking. As demonstrated in Fig. S5A, the G' and G'' of the GTU-Fe hydrogel are significantly higher than those of other hydrogels owing to the superimposition effect of the quadruple hydrogen bond between UPy molecules and the complexation of iron and carboxyl groups. The values of G' and G'' remain stable within a strain range of 0.1-300% and a shear rate range of 0.1-10 Hz, indicating mechanical stability of the GTU-Fe hydrogel. Interestingly, the GTU-Fe hydrogel exhibits a self-healing property. As depicted in Fig. S5C, the hydrogel network is immediately restored to its original state when a strain of 1% or 1,000% is applied), suggesting that the hydrogel remains stable under dynamic conditions.

As shown in Fig. 3B, the GTU-Fe hydrogel, due to its unique design, aesthetic configuration, and easy operation, can be molded into various patterns and then rolled into a cylindrical hydrogel scaffold. This scaffold can instantly recover to its original shape upon removal of the external compression force (Fig. 3C and Movie S1). It also possesses a compressive strength of 2.59 MPa (Fig. S6A) and excellent fatigue resistance to cyclic compression tests (Fig. S6B). The self-healing property of the GTU-Fe hydrogel was confirmed by a mechanical test. As shown in Fig. S6C, the GTU-Fe hydrogel exhibits a rapid self-healing response after being cut into small fragments. The tensile strength of the GTU-Fe hydrogel returns to its initial value after healing (Fig. S6D).

As illustrated in Fig. 3D, a micro-CT scan was used to examine the groove structure of the resulting hydrogel. The grooves in the hydrogel film act as reservoirs to separately hold KGN@PDA and miRNA@CaP. GTU-Fe hydrogel exhibited excellent adhesion property and NIR-assisted roll-film capability (Fig. S7). A cylindrical scaffold was effectively formed by rolling the patterned GTU-Fe hydrogel film into an osteochondral plug irradiated by NIR for 3 min. The EDS spectrum confirmed that the hydrogel pattern consisted of a gully structure (Fig. 3E). The SEM images (Fig. 3F, G) reveal that the surface of the GTU-Fe hydrogel is covered with uniformly sized NPs. The EDS spectrum validates that KGN@PDA and miRNA@CaP are deposited in situ onto the hydrogel surface (Fig. S8A). The full survey scans of the XPS spectra are presented in Fig. 3F.

New peaks for calcium (Ca 2p) and phosphorus (P 2p) appear alongside four intense peaks (O 1s, N 1s, C 1s and Fe 2p), indicating successful integration of miRNA@CaP onto the surface (Fig. 3H, I). Owing to the complexation with phenolic hydroxyl groups, the binding energy of iron (Fe) increases, as presented in Fig. 3J. As shown in Fig. 3K, the binding energy of C-OH is 531.10 eV in the GTU-Fe sample, but increases to 531.73 eV after loading KGN@PDA. This shift signifies a decrease in the density of the electron cloud around C-OH due to the complexation of Fe^3+^ with C-OH. The binding energy of C=O increases from 532.18 to 532.40 eV in GTU-Fe/miRNA@CaP. The lone pair electrons of oxygen (O) in the carbonyl group interact with Ca^2+^, resulting in a decrease in the density of the electron cloud around C-OH. These observations suggest that the carbonyl groups play a coordinating role with Ca^2+^ to form coordination compounds in the GTU-Fe hydrogel. The XPS data confirm that KGN@PDA and miRNA@CaP are spatially deposited in situ on the patterned hydrogel.

Numerous free radicals such as reactive oxygen species (ROS) are often concentrated at the site of cartilage-bone defects, inflicting substantial damage to the lesion area [29]. Excessive accumulation of ROS in wound tissue has been proven to promote the expression of pro-inflammatory factors, restricted angiogenesis, and disrupted collagen deposition [30]. Exceptional free radical scavenging ability can reduce inflammation reactions during in vivo application. Here, we used DPPH, ABTS, and PTIO· to assess the capacity of the hydrogel scaffold to scavenge free radicals. As shown in Fig. S9, the GTU-Fe/KGN@PDA/miRNA@CaP hydrogel scaffold can capture 91% of DPPH within 60 min, 39% of ABTS within 6 min, and 16% of PTIO· within 120 min. The introduction of PDA enhanced the free radical scavenging ability of the GTU-Fe/KGN@PDA/miRNA@CaP hydrogel scaffold, thereby reducing the in vivo inflammatory response. The observed differences result from the varied types of free radicals used in the characterization methods. DPPH and ABTS are nitrogen-based free radicals, while PTIO· is an oxygen-based free radical, which better represents the ROS in organisms and exhibited the highest biological correlation. The hydrogel scaffold displayed strong ROS scavenging ability based on the PTIO· method results (Fig. S9).

As depicted in Fig. S10, on the first day, the cumulative release rate rapidly increases with incubation time and slows significantly over the next 7 days. After 7 days of incubation, 96% of KGN had diffused from the dialysis bag into the PBS solution. Both KGN and miR-26a demonstrated the same drug release pattern. The cumulative release rate of miR-26a was fast on the first day and reached approximately 55% after 7 days of incubation (Fig. S10).

### Effect of KGN and miR-26a on MSCs/Chondrocytes

Cell growth curves indicated that a time-dependent growth of MSCs over 1 and 3 days of culture. The viability of cells showed significant differences between MSCs treated with and without KGN or miR-26a (*p*<0.05). MSCs treated with KGN at 100 nM showed higer level of proliferation, than that of MSCs treated with KGN at concentrations of 25, 50, 200, and 400 nM in day 1. (*p*<0.05) (Fig. S11), similar trends were observed in chondrocytes (Fig. S12). Chondrocytes treated with miR-26a at 50 nM showed significantly better proliferation than those treated with miR-26a at concentrations of 12.5, 25, 100, and 200 nM in day 1 (*p*<0.05) (Fig. S12), but MSCs treated by miR-26a with different concentration did not indicate distinct statistical differences at the same time (Fig. S11). Similar trends were observed in chondrocytes. Therefore, we opted to use 100 nM of KGN and 50 nM of miR-26a for subsequent migration and differentiation studies.

The cytotoxicity and cytocompatibility of GTU-Fe/miRNA@CaP and GTU-Fe/KGN@PDA were evaluated using the CCK-8 assay with MSCs and chondrocytes. Culturing MSCs with the scaffolds for 1 and 3 days did not impact cell proliferation or viability compared to the control (Fig. S11). Similar observations were made when chondrocytes were cultured with the scaffolds (Fig. S12). These results confirm the non-cytotoxicity and good cytocompatibility of the hydrogel scaffolds.

Cell migration within or into artificial wounds was observed over a 12h period. After 3, 6, and 12 h the closed areas of MSCs in the KGN and miR-26a groups were similar to the control group (Fig. S13), suggesting that the migration ability of MSCs was not significantly affected by the two molecules at the experimental concentrations.

**Fig. 4:**
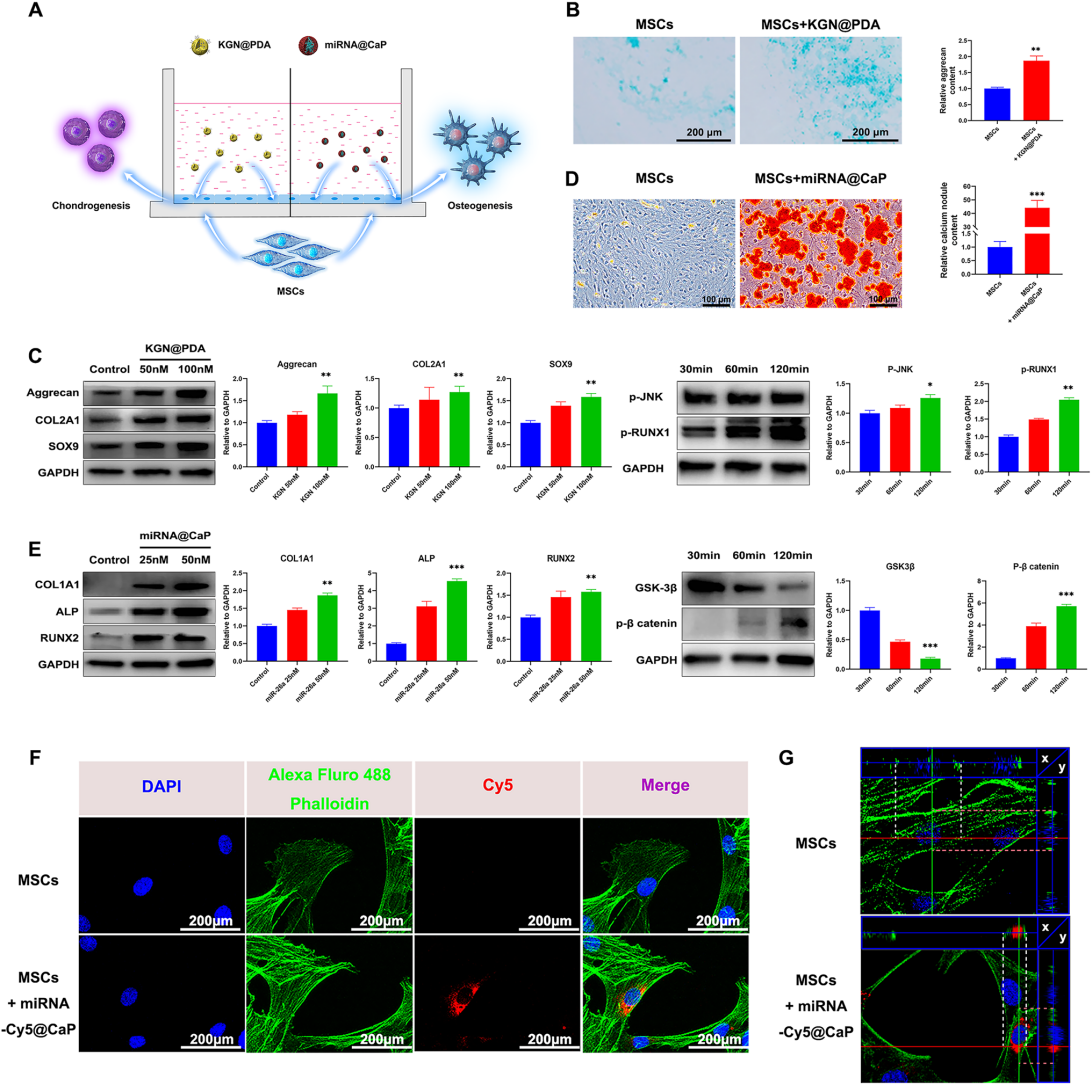
In vitro evaluation of the effect of KGN@PDA and miRNA@CaP NPs on the chondrogenesis and osteogenesis of MSCs respectively and the cellular uptake of miR-26a in MSCs. **A** Scheme of MSCs co-culture system  with KGN@PDA or miRNA@CaP in vitro. **B** Chondrogenic differentiation was stained by Alcian blue and quantitatively analyzed at day 14. Scale bar =200 m. **C** Western blot analysis of Aggrecan, COL2A1 and Sox9 expression levels in MSCs induced by KGN@PDA (50 nM, 100 nM) for 14 days and p-JNK, p-RUNX1 expression levels for 30, 60, 120 min and quantification of protein expression. **D** Osteogenic differentiation was detected by Alizarin Red staining at 7 days and quantification of calcium nodules. Scale bar=100 m. **E** Western blot analysis of COL1A1, ALP and RUNX2 expression levels in MSCs induced by miRNA@CaP (25 nM, 50 nM) for 7 days and GSK-3, p- catenin expression levels for 30, 60, 120 min and quantification of protein expression. **F** Laser confocal photographs of cellular uptake of miR-26a-Cy5 in MSCs (Cytoplasm: green; Nucleus: blue; Cy5: red). Scale bar=200 m. **G** Sectional images of cell in the control and experimental groups. Data are presented as mean±SD (*n*=3)

The impacts of KGN@PDA and miRNA@CaP on the chondrogenic and osteogenic differentiation of MSCs were assessed using a co-culture system (Figs. 4A and S14)KGN@PDA significantly enhanced the secretion of cartilage ECM, indicating chondrogenic differentiation (Fig. 4B), the protein expressions of Col II, Sox9, and aggrecan in MSCs were upregulated, concurrently, the upregulation of protein p-JNK and p-RUNX1 was observed, suggesting that KGN promotes chondrogenic differentiation through the JNK/RUNX1 pathway (Fig. 4C).

Contrastingly, miRNA@CaP induced notable osteogenic differentiation of MSCs, as indicated by increased expression of calcium nodules, protein COL1A1, ALP and RUNX2 with escalating concentrations of miR-26a (25-50 nM) (Fig. 4D, E). The downregulation of GSK-3β protein expression and the upregulation of p-β catenin suggest that miR-26a promotes osteogenic differentiation via the GSK-3β/β-catenin pathway (Fig. 4E).

To examine the cellular uptake of miR-26a in monolayer culture, Cy5 was chosen as the fluorescent dye. After co-culturing miRNA-Cy5@CaP with MSCs for 1 day, fluorescent microscopy images displayed high-intensity fluorescence signals within the cytoplasm (Fig. 4F). To further investigate whether miRNA@CaP was intracellular rather than extracellular, 3D reconstruction was performed and sectional images were obtained (Fig. 4G). It was observed that the nucleus was surrounded by miR-26a, which was primarily distributed on the interior side of the cell membrane. This result indicates that miR-26a can be effectively transfected into MSCs in the early stages and uniformly distributed within the cytoplasm.

### Microarray and Analysis

**Fig. 5:**
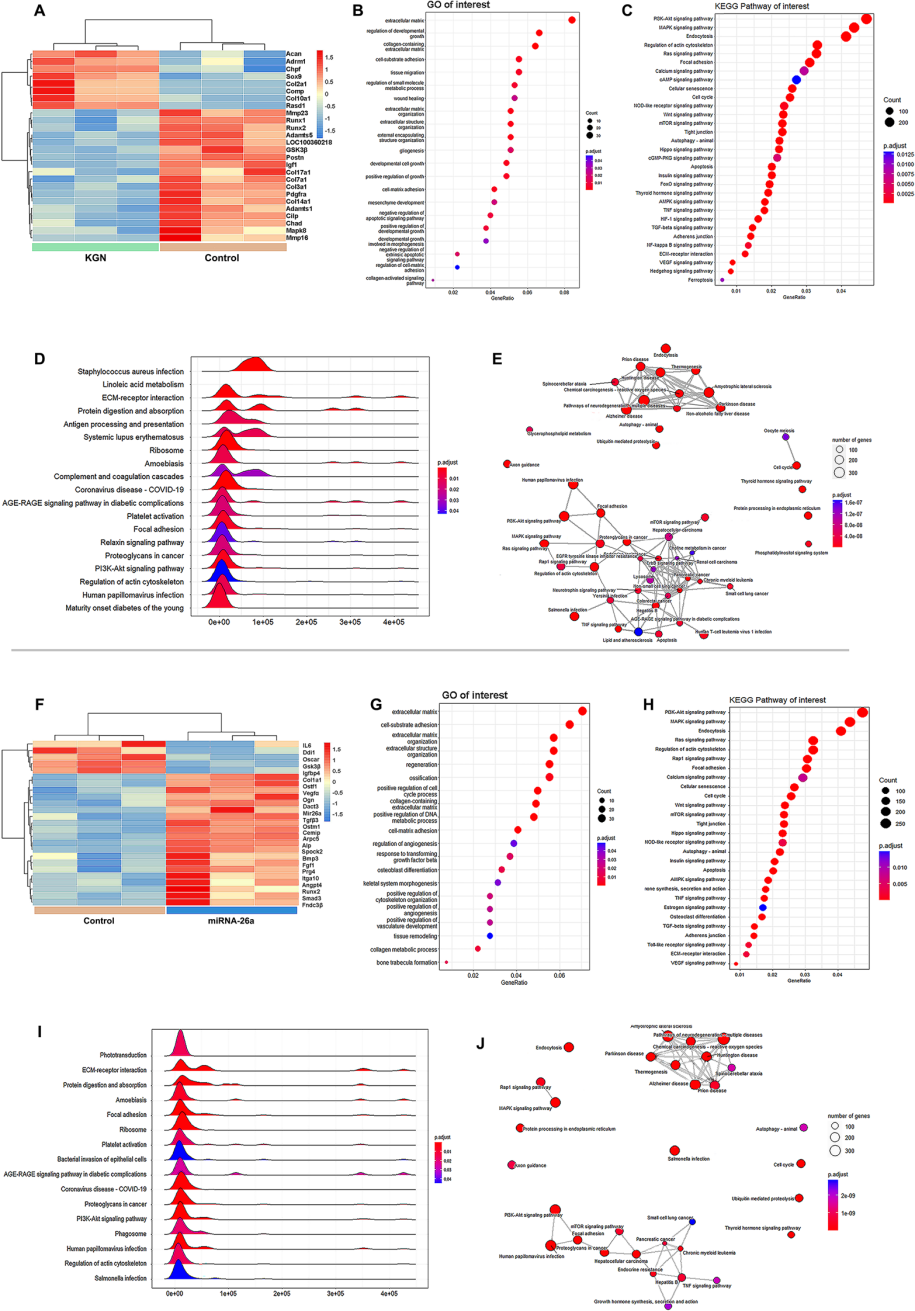
RNA-Seq-based transcriptome analysis of chondrogenesis or osteogenesis of MSCs in vitro. Heatmap cluster analysis showed relative expression levels for the differentially expressed genes treated by **A** KGN or **F** miR-26a. Gene ontology (GO) analysis for differentially expressed genes of MSCs transcriptome treated by **B** KGN or **G** miR-26a. Enrichment analysis of interested Kyoto Encyclopedia of Genes and Genomes (KEGG) pathway treated by **C** KGN and **H** miR-26a. Ridgeplot of GSEA analysis treated by **D** KGN or **I** miR-26a. KEGG pathway interaction diagram after treated by **E** KGN or **J** miR-26a

RNA microarray analysis was conducted to explore the gene expression profiles of MSCs treated with KGN or miR-26a. Selected differentially expressed genes (DEGs) underwent cluster analysis. After KGN treatment, the upregulation of COL2A1, SOX9, ACAN, and the downregulation of MMP23 and ADAMTS5, indicated upregulated chondrogenic differentiation and diminished ECM degradation (Fig. 5A). Concurrently, the upregulation of COL1A1, ALP, and RUNX2 suggests miR-26a-induced osteogenic differentiation (Fig. 5F). GO analysis demonstrated that collagen-containing ECM, developmental cell growth, and negative regulation of the apoptotic signaling pathway are involved in chondrogenesis (Fig. 5B). In contrast, ECM organization, ossification, and regulation of angiogenesis are implicated in osteogenesis (Fig. 5G). KEGG pathway enrichment analysis revealed significant enrichment of the PI3K-Akt, MAPK, and endocytosis pathways in both the KGN-induced chondrogenesis (Fig. 5C) and miR-26a-induced osteogenesis (Fig. 5H) groups. Gene set enrichment analysis (GSEA) of the KEGG pathway identified 19 pathways involved in chondrogenesis (Fig. 5D) and 16 pathways involved in osteogenesis (Fig. 5I). Pathways including the PI3K-Akt signaling, ECM-receptor interaction, focal adhesion, and regulation of the actin cytoskeleton pathways were significantly enriched. Figure 5E, J displays the KEGG pathway interaction diagrams.

### In Vivo Evaluation

We first evaluated the biosafety of GTU-Fe, GTU-Fe/KGN@PDA, and GTU-Fe/miRNA@CaP through histopathological analysis of primary organs and the inflammatory response post-subcutaneous implantation. No severe inflammation reactions were detected in any of the groups (Fig. S15). Simultaneously, the biotoxicity of nanoparticles to different organs was studied by hematoxylin eosin (H&E) staining [31]. The results indicated that H&E-stained heart, liver, spleen, lung, and kidney samples revealed no discernible changes following treatment with GTU-Fe, GTU-Fe/KGN@PDA, or GTU-Fe/miRNA@CaP (Fig. S16). Furthermore, blood biochemistry indicated that the hydrogel had no biotoxic or side effects (Fig. S17).

After a week of post-hydrogel implantation into the osteochondral defects, we examined the knee joints of each rabbit. No obvious signs of inflammation such as redness, swelling, fever, or pain were observed. Moreover, no rabbits succumbed to postoperative infection before sacrifice.

Red fluorescence in the cartilage defect zones was detected using a small animal in vivo fluorescence imaging system at 6 and 12 weeks post-implantation, confirming the presence of miR-26a (Fig. S18A). However, the fluorescence intensity gradually weakened in a time-dependent manner. After performing frozen sectioning, red fluorescence remained visible in the defect zones under confocal microscopy (Fig. S18B).

**Fig. 6:**
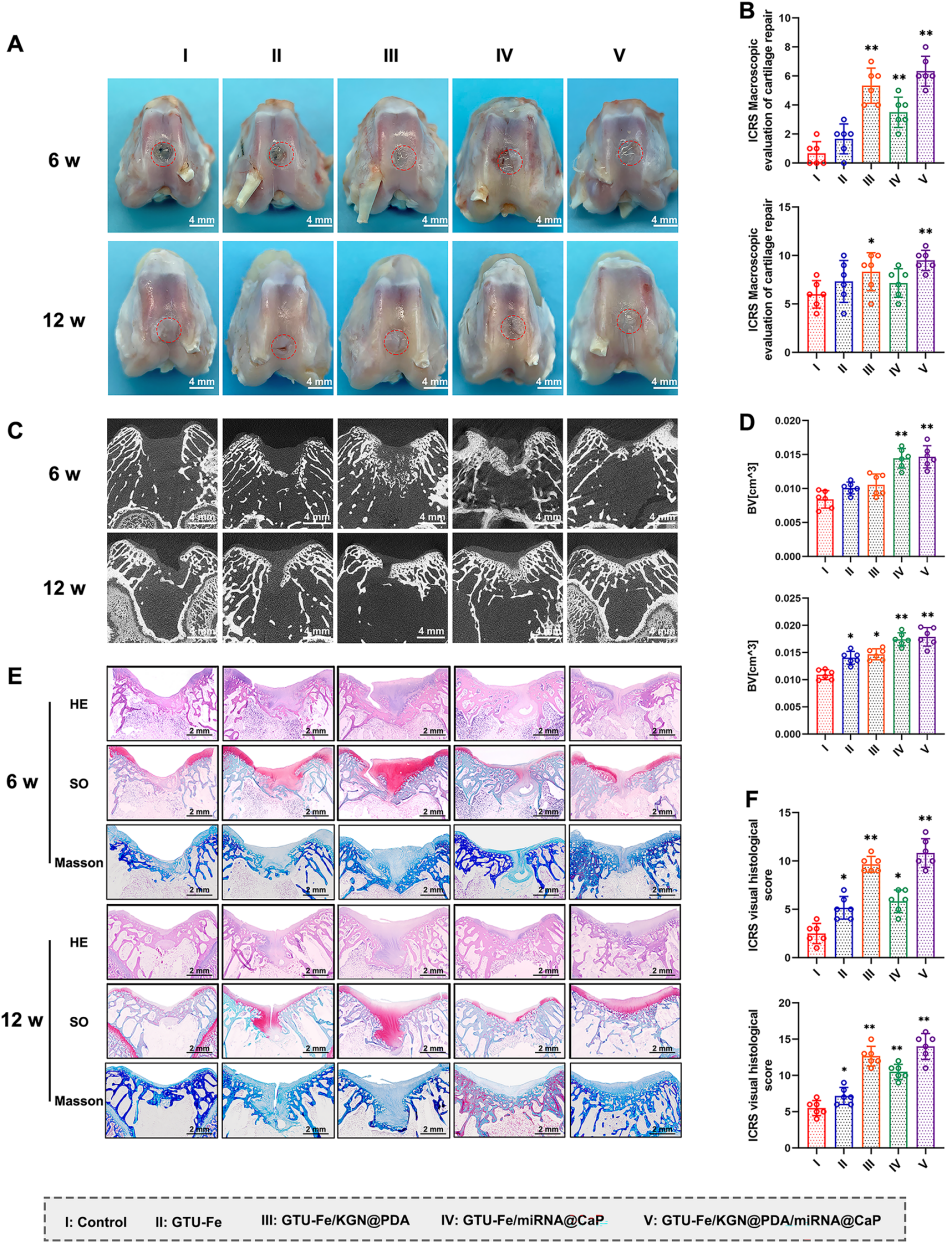
Macroscopic assessment of in vivo repair effect. **A** Gross morphology of formed tissues treated with different engineered implants after 6 and 12 weeks. Scale bar = 4 mm. **B** ICRS macroscopic scores of articular cartilage for the different groups. **C** Representative micro-CT coronal plane images and **D** quantitative analysis of bone regeneration in the regenerative osteochondral defects (BV, the bone volume). Scale bar = 4 mm. **E** Representative images of H&E, Safranine O (SO) and Masson staining of the cartilage defects at 6 and 12 weeks post-surgery. Scale bar = 2 mm. **F** ICRS visual histological scores of repaired cartilage of the different groups. The p-value was calculated by one-way ANOVA test (*p < 0.05 and **p < 0.01). Data are represented as mean ± SD (*n* = 6)

Six-week post-surgery, the defects in all groups remained distinguishable from the surrounding tissue. Group I displayed unfilled defects, while Groups II and III showed partial filling. In Groups IV and V, the defect margins were obscure. However, as shown in Fig. S19, at 12 weeks, Group V defects were covered with shiny, smooth white tissue, reminiscent of articular cartilage. The defect size in Group I had diminished. In the remaining groups, the defects were also filled with smooth white tissue, but the borders between the regenerated tissue and surrounding cartilage were evident, especially in Groups II and III (Fig. 6A). The macroscopic data and gradings of repaired articular cartilage are displayed in Fig. 6B. Group V scored the highest macroscopic scores at both 6 and 12 weeks post-surgery (n = 6, *p* < 0.05) (Fig. 6B).

Micro-CT images were used to track the formation of new subchondral bone. At 6 weeks, more calcified tissue formed in Groups IV and V, surpassing Groups III, II, and I in the volume of calcified tissue (Figs. 6C and S20). Group I demonstrated the least subchondral BV. At 12 weeks, Group V exhibited the most pronounced subchondral bone formation, with the newly formed bone almost filling the entire subchondral region. The repair effect of Group IV also improved, albeit less than that of Group V. Groups II and III also revealed abundant subchondral bone formation, albeit significantly less than that of Groups IV and V. However, in Group I, scant subchondral bone was detectable (Figs. 6C and S20). Group V had the highest BV among all groups (Fig. 6D), underscoring the excellent osteogenic ability of miR-26a.

At 6 weeks, histological observation indicated negligible cartilage regeneration in Group I’s defect. Group II’s defect section was partially filled with cartilage tissue, but the cartilage matrix was scant. Group III’s defect contained an abundant cartilage matrix. Group IV displayed considerable subchondral bone regeneration and a sparse cartilage matrix. Group V demonstrated superior subchondral regeneration and cartilage regeneration. The cartilage matrix, indicated by Masson’s trichrome and Safranin-O staining, showed a small amount of collagen and GAG, signifying ongoing cartilage matrix production. No residual hydrogels were observed, indicating that the hydrogels had almost completely degraded (Fig. 6E).

At 12 weeks, Group I still had a discontinuous section, and the defect was filled with thin, fibrous cartilage tissue. Groups II and III exhibited an increased cartilage matrix. Subchondral bone formation improved in Group IV, and an abundant cartilage matrix was regenerated in Group V, albeit with discontinuities in the neocartilage. Group V demonstrated the best cartilage regeneration, with the defects primarily filled with white neocartilage. This continuous neocartilage was similar to normal cartilage, with abundant cartilage matrix and collagen arrangement consistent with that of the normal cartilage. Complete subchondral bone reconstruction was observed (Fig. 6E). Group V had the highest ICRS scores at both 6 and 12 weeks post-surgery (all *p* < 0.05) (Fig. 6F).

**Fig. 7:**
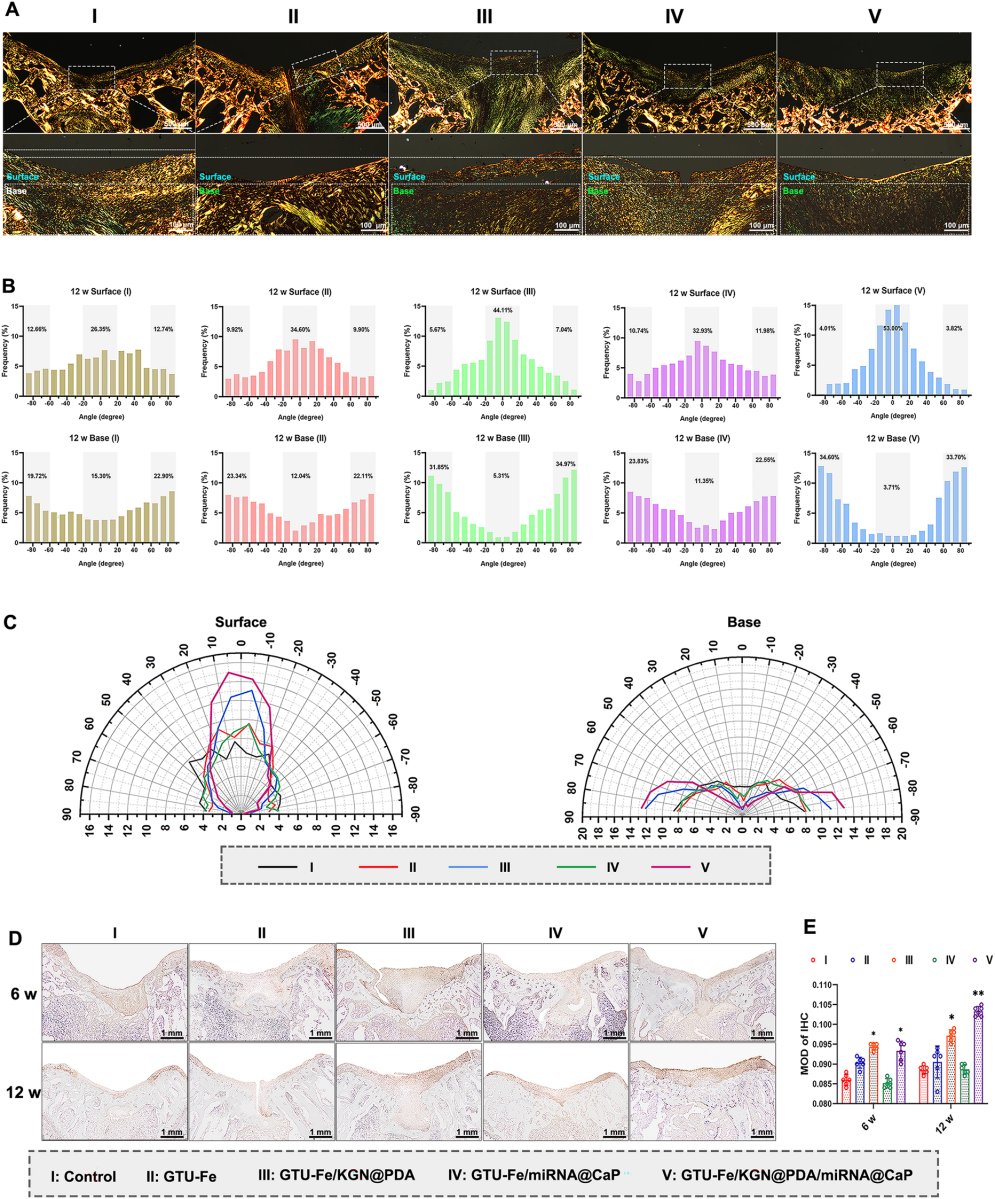
**A** Picrosirius red staining of regenerated tissue at 12 weeks post-surgery. Scale bar: upper, 500 µm; lower, 100 µm. **B** Quantitative analysis of collagen fiber distribution of the repaired tissue in the surface area (upper 1/3) and base area (bottom 2/3). **C** Comprehensive comparison of collagen orientation within surface and base areas between control, GTU-Fe, GTU-Fe/KGN@PDA, GTU-Fe/miRNA@CaP, and GTU-Fe/KGN@PDA/miRNA@CaP groups presented by polar coordinates. **D** Representative images of immunohistochemistry staining of Col II of the cartilage defects at 6 and 12 weeks post-surgery. Scale bar = 1 mm. **E** Quantification of Col II expression for the different groups. The p-value was calculated by one-way ANOVA test (*p < 0.05 and **p < 0.01). Data are represented as mean ± SD (*n* = 6)

Polarized light microscopy revealed an increase in collagenous fiber levels from 6 to 12 weeks across all groups (Figs. S21 and 7A). However, at 12 weeks, the collagenous fibers within the repaired tissues of Group I were disorganized. In Groups II and IV, the collagen fibers were more regular. Group III had abundant fibers, while Group V boasted rich and nearly vertical collagen fibers, similar to native cartilage. The birefringence (an indicator of collagen content) of repaired cartilage was marginally weaker than that of the surrounding cartilage. The birefringence of subchondral bone in the repaired areas and adjacent tissues in Groups IV and V were similar (Fig. 7A).

The collagen distribution within the repaired issues is illustrated in Figs. S21 and 7B, C. At 12 weeks, Group I showed no difference of collagen distribution between the surface and the base zones, suggesting that only fibrous cartilage was formed. In Group II, collagen fibers in the surface zone primarily oriented between  20° and 20°, accounting for 34.6% of all fibers, compared to 26.4% in Group I. In the base zone, collagen alignment was more directed toward ± 60°– ± 90° in Group II, reaching up to 45.4% compared to 42.6% in the blank group. These results improved further in Group III and peaked in Group V, with 44.1% and 53.0% of parallel collagen in the superficial zone, and 66.8% and 68.3% in the base zone, respectively (Fig. 7B, C).

Abundant Col II staining was observed in Groups III and V, with the Col II level increasing from 6 to 12 weeks. In the control group, Col II was predominantly confined to the borders of host cartilage and the defect bases. Group II and Group IV displayed more intense Col II staining than Group I, particularly at the interface. Neocartilage was flawlessly integrated with subchondral bone in Group V. The quantitative analysis of positive Col II revealed that Group V contained significantly more Col II than the other groups (Fig. 7D, E).

To identify the relative expression levels of COL1A1, ALP, COL2A1, aggrecan, and SOX9, regenerative osteochondral tissues were collected post-sacrifice and the relative miRNA level in each group was determined by qRT-PCR. Group III and V exhibited significantly higher expression of Col2A1, aggrecan, and SOX9 at 6 and 12 weeks than the other groups (all *p* < 0.05). Groups IV and V also showed higher expression of Col1A1 and ALP (Fig. S22).

### Discussion

In this study, a patterned supramolecular hydrogel was fabricated as the osteochondral scaffold to in situ deposit KGN@PDA and miRNA@CaP. This hydrogel demonstrated successful comprehensive repair of bone and cartilage, maintaining long-term repair effects and reducing deterioration in vivo. These desirable outcomes were attributed to the stable mechanical properties, self-healing properties, high ROS capture ability, and controllable release of KGN and miR-26a. The newly formed cartilage and subchondral bone exhibited structural and functional similarity to native cartilage, indicating well-integrated tissue regeneration. This biomaterial-guided drug and gene delivery system [32] constitutes a novel approach to regulate cell behavior spatiotemporally by loading different factors in specific zones and mimicking the natural tissue structure. Its potential applications extend beyond osteochondral repair, offering a promising solution for the repair of multiple tissue types in the future.

To date, numerous studies have focused on the development of novel scaffolds that mimic the cartilage ECM [33, 34]. Previous research has shown that oriented scaffolds resembling the native tissue structure have the potential to engineer specific tissues by enhancing cell adhesion and proliferation [35, 36]. The longitudinal microtubular orientation of scaffolds can regulate cell migration and mass transportation, influencing the thickness and homogeneity of in vitro and in vivo cartilage formation [37, 38]. In our study, the oriented hydrogel demonstrated promise in cartilage repair, exhibiting increased collagen and GAG deposition compared to the control group. The orientation of the scaffolds effectively enhanced the thickness and homogeneity of cartilage. The parallel orientation of microtubules facilitated efficient nutrient and metabolite transport to the inner part, while promoting homogeneous cell distribution and effective mass transportation, resulting in simultaneous cartilage formation in both outer and inner parts; this contributed to the regeneration of thick and homogeneous cartilage.

KGN is a non-protein small molecule known for inducing chondrogenic differentiation. While direct injection of KGN resulted in significant improvement in tissue repair for cartilage defects [39], in vivo studies have demonstrated that sustained delivery systems using small molecule therapy have greater potential for cartilage regeneration [13] and even for treating osteoarthritis (OA) [16]. In our study, Group III exhibited abundant GAG and Col II content in the cartilage defects. KGN preconditioning likely enhanced the chondrogenic differentiation of MSCs by promoting their commitment to a precartilaginous stage through enhanced JNK phosphorylation and suppressed β-catenin signaling [40]. The sustained release of KGN from the hydrogels not only promoted chondrogenic differentiation of MSCs, but also significantly inhibited hypertrophy, as reported previously [41]. The long-term release of KGN efficiently and persistently activated multiple genes and signaling pathways, promoting chondrogenesis and resulting in regenerated tissues with well-matched histomorphology and biomechanical performance. Additionally, KGN protected the expression of cartilage matrix components such as Col II and aggrecan in IL-1β-stimulated chondrocytes, indicating its potential to promote cartilage matrix synthesis in an inflammatory environment [42].

Previous studies reported abnormal expression of certain miRNAs (miR-133, miR-135, miR-138, miR-637, and miR-26a) during osteogenic differentiation of MSCs, suggesting their involvement in bone repair [43, 44]. Li et al. [21] investigated the delivery of MSCs transfected with miR-26a-loaded hydrogel in a mouse calvarial defect model, demonstrating the bone repair-promoting effects of miR-26a, which regulates angiogenesis-osteogenesis coupling. The interconnectivity between vasculature formation and bone regeneration deserves increasing attention in tissue engineering [45]. The use of 3D hybrid nanofiber aerogels loaded with miR-26a also had enhanced the healing of cranial bone defects [46]. Local administration of miR-26a-5p was found to accelerate femur fracture healing in mice [47]. Consistent with previous studies [48], our results demonstrated that delivery of miR-26a led to reduced expression of GSK-3β, further confirming its osteogenic role in vitro. The cumulative release rate of miR-26a was approximately 55% after 7 days of incubation, enhancing osteogenesis through the downregulation of GSK-3β. miR-26a targets and inhibits GSK-3β, thereby promoting osteogenic differentiation [49, 50]. Moreover, upregulating the expression of miR-26a has been shown to attenuate cartilage injury, stimulate chondrocyte proliferation, and inhibit apoptosis in rats with rheumatoid arthritis (RA) [51]. miR-26a has also been demonstrated to increase vascularization and coordinate the coupling of angiogenesis and osteogenesis, which is crucial for neo-bone maturation [46]. miR-26a actively participates in the osteogenic differentiation of MSCs, enhancing ALP expression [52, 53]. The evaluation of these scaffold-based miR-26a applications to enhance bone formation in vivo is commonly performed through micro-CT analysis, which assesses bone bridging, new BV, as well as bone area and mineral density, as shown in Fig. 6 [54]. The reconstruction of subchondral bone was significantly improved in the GTU-Fe/miRNA@CaP group.

Stable bone ingrowth at the bottom (bone side) of the implant is crucial for providing appropriate mechanical support to the developing overlying cartilage. The capacity of articular cartilage to heal when separated from the subchondral bone is limited. In cases of delaminated cartilage in the knee joint, even without a transchondral or flap tear, it has been recommended that the cartilage be debrided as it will not reattach to its bony bed [55]. Previous studies have also emphasized the importance of healthy subchondral bone in articular cartilage repair [56], which aligns with the findings of the present study.

Given the positive effects of KGN on cartilage and miR-26a on bone, our results indicate that the delivery of KGN and miR-26a yielded a better approximation of the normal zonal collagen network and subchondral regeneration compared to the other groups. The restored collagen fibrils induced by KGN treatment, mimicking the primary vertical orientation in the base zone of normal cartilage, may significantly enhance tissue stiffness and protect the solid matrix against large distortions and strains at the subchondral junction [57]. This effect could potentially contribute to the persistent and enhanced repair of cartilaginous tissue. Histological observations and quantification of GAG and Col II content confirmed significantly higher deposition in Group V compared to the other groups, supporting the gene expression findings. Group V demonstrated improved quality of repaired cartilage, as evidenced by histological staining, and better subchondral bone reconstruction as assessed by micro-CT. The histological staining of Group V revealed more hyaline-like cartilage in terms of ECM, cartilage lacuna, and type II collagen [58]. Visual evaluation of collagen orientation and distribution indicated better alignment and distribution of collagen fibers, primarily directed toward the subchondral bone plate in the basal zone of Group V compared to the other groups. Moreover, Group V exhibited a significantly larger area of cartilage and subchondral regeneration compared to the other groups. Collectively, these findings demonstrate that KGN/miR-26a treatment via oriented hydrogel-guided delivery significantly improved major parameters of stratified zonal in situ chondrogenesis and osteogenesis.

## Conclusions

In this study, we drew inspiration from the natural Haversian canal structure of bone and employed a film-rolling approach to fabricate an oriented osteochondral scaffold hydrogel. A significant innovation was the in situ deposition of both KGN and miR-26a on a patterned hydrogel matrix through dynamic and responsive coordination complexation between metal ions and their ligands. Our meticulously designed osteochondral plug exhibited remarkable capacity to promote the restoration of osteochondral defects in a rabbit model, leading to the successful integration of neocartilage with the subchondral bone. This biomaterial-guided delivery approach represents a significant advance toward enhancing osteochondral repair and achieving seamless integration in the near future.

## Supplementary Information

Below is the link to the electronic supplementary material.

Supplementary file1 (PDF 3807 kb)

Supplementary file2
