# Supplementary material for: In Situ Deposition of Drug and Gene Nanoparticles on a Patterned Supramolecular Hydrogel to Construct a Directionally Osteochondral Plug
Source: Nanomicro Lett. 2023 Nov 17;16:18. doi: 10.1007/s40820-023-01228-w (PMC10656386; doi:10.1007/s40820-023-01228-w)
Supplement: Supplementary file 1 — Supplementary file1 (PDF 3807 kb) [file 40820_2023_1228_MOESM1_ESM.pdf]

Supporting Information for

In Situ Deposition of Drug and Gene Nanoparticles on a Patterned Supramolecular Hydrogel to Construct a Directionally Osteochondral Plug

Jiawei Kang [1, †], Yaping Li [2,3,†], Yating Qin [2,3], Zhongming Huang [4], Yifan Wu [1], Long Sun [5], Cong Wang [1], Wei Wang [2,3,]*, Gang Feng [1,]*, Yiying Qi [1,] *

[1] Department of Orthopaedic Surgery, the Second Affiliated Hospital, Zhejiang University School of Medicine, Hangzhou City, 310009, Zhejiang Province, P. R. China

[2]College of Chemical and Biological Engineering, Zhejiang University, Hangzhou 310027, P. R. China

[3] ZJU-Hangzhou Global Scientific and Technological Innovation Center, Hangzhou, Zhejiang 311215, P. R. China

[4]The Affiliated Nanhua Hospital, Orthopedic Research Centre, Hengyang Medical School, University of South China, Hengyang, Hunan, 421001, P. R. China

[5] Department of Radiology, Jining No. 1 People's Hospital, Jining Medical University, Jining 272000, Shandong, P. R. China

† Jiawei Kang and Yaping Li contributed equally to this work.

*Corresponding authors. E-mail: wwgfz@zju.edu.cn (Wei Wang); gangfeng@zju.edu.cn (Gang Feng); qiyiying@zju.edu.cn (Yiying Qi)

S1 Experimental

S1.1 Materials and Reagents

2-Amino-4-hydroxy-6-methylpyrimidine, 1,6-diisocyanatohexane, dimethyl sulfoxide (DMSO), anhydrous ferric chloride ($FeCl_3$), sodium citrate (Na_3Cit), and disodium hydrogen phosphate (Na_2HPO_4) were procured from Shanghai Aladdin Biochemical Technology Co., Ltd. (Shanghai, China). Gelatin (type A), KGN, dopamine hydrochloride (DA), calcium chloride ($CaCl_2$), and tris(hydroxymethyl)aminomethane hydrochloride were supplied by Sigma-Aldrich (St. Louis, MO, USA). Sodium hydroxide (NaOH) was sourced from Sinopharm Chemical Reagent Co., Ltd. (Shanghai, China). Hsa-miR-26a was provided by Guangzhou RiboBio Co., Ltd. (Guangzhou, China). Alexa Fluor® 488 phalloidin was purchased from Thermo Fisher Scientific Co., Ltd. (Shanghai, China). Modified Saffron-O and Fast Green Stain Kit (For Bone) was purchased from Solarbio LIFE SCIENCES Co., Ltd. (Beijing, China). All these reagents were used without any additional purification.

S1.2 Preparation and Characterizations of GTU Hydrogel

Gelatin-UPy was used as the matrix to in situ load the resulting NPs. Gelatin-UPy was synthesized through a typical two-step method as per previous reports [S1]. Initially, 2-amino-4-hydroxy-6-methylpyrimidine (6 g) and 1, 6-diisocyanatohexane (55 g) were added to a reaction flask and heated to 100 ºC for 16 hours under a nitrogen atmosphere. Afterwards, 200 mL of n-pentane was added to the flask for precipitation. The precipitate was filtered under reduced pressure, washed three times with n-pentane, and then dried in a vacuum at 50ºC for 24 hours to produce UPy-NCO.

In the second step, 5 g of gelatin was dissolved in 50 mL of anhydrous DMSO at 80ºC under a nitrogen atmosphere. Once the gelatin was fully dissolved, the solution was cooled to room temperature. Then, 0.5 g of UPy-NCO/DMSO was added dropwise to the gelatin solution. The mixture was stirred overnight at 25ºC under nitrogen, followed by precipitation in ethanol three times. The precipitate was filtered, extracted with ethanol, and finally dried under vacuum at 50 ºC for 24 hours to obtain gelatin-UPy. The UPy grafting ratio could be adjusted by the mass ratio of gelatin and UPy-NCO and determined by the ninhydrin color reaction as reported previously [S2, S3]. GTU with the feeding ratio 5% and 15% presented worse mechanical properties and weak water solubility, respectively. GTU with grafting rate of 6.12% (10% feeding ratio) was chose for the further study.

The resultant gelatin-UPy was measured using a 400 MHz nuclear magnetic resonance instrument (^{1}H-NMR; Avance-400; Bruker), an attenuated total reflectance-infrared (ATR-IR) system (NICOLET iS50FT-IR; Thermo Fisher), and ultraviolet-visible (UV-vis) spectroscopy (GENESYS 180; Thermo Fisher).

The GTU-Fe hydrogel was prepared by mixing a GTU solution (20 wt%) with a $FeCl_3$ solution (12 mM). This mixture was then homogenized using a vortex (Vortex-6; HaiMen Kylin-bell Lab Instrument, Jiangsu, China) at 3,000 rpm for 10 seconds. The resulting hydrogel precursor solution was poured into a customized polytetrafluoroethylene (PTFE) mold (Lishizhuangong-L6, BSXCNC, shenzhen, China) and left for 24 hours, then removed from PTFE mold acquire the patterned hydrogel film. Gelatin-Fe hydrogel was also prepared following the same procedure, except that gelatin was used to replace gelatin-UPy, which is referred to as G-Fe.

Dynamic rheological measurements of various hydrogel samples were conducted using a rheometer (DHR-2; TA, USA). The linear viscoelastic region was determined by fixing the frequency at 1 Hz over a strain range of 0.1%-1,000%. The gel strength was calculated based on the aforementioned strain-sweep tests. Frequency-sweep tests were performed at 1% strain over a frequency range of 0.1-100 Hz. The self-healing performance was measured by employing the alternating step strain scanning method (1% or 1,000%).

The compression performance of the hydrogels was tested on a computerized electronic universal testing machine (UTM2102; SUNS, Shenzhen, China). For loading-unloading tests, the hydrogel patch was shaped into a cylinder (10 mm in diameter and 20 mm in height), and five successive loading-unloading compressive tests were conducted on these cylindrical hydrogels at a fixed strain of 50%. The speed was set at 5 mm min^{-1}. The tensile properties of both the hydrogel and the self-healed hydrogel were examined. The sample was cut into a rectangle (50 mm in length, 10 mm in width, and 2 mm in thickness) and subjected to a tensile speed of 10 mm min^{-1}.

A dopamine buffer solution was prepared according to the aforementioned step, and KGN NPs aqueous dispersion was added to this solution and transferred to the groove of the patterned hydrogel film. KGN@PDA NPs were then synthesized in situ on the patterned hydrogel film (3 mm in width, 13 mm in length and 1 mm in height). The miR-26a/$CaCl_2$ solution and Na_2HPO_4 solution were mixed and quickly transferred to the groove of the patterned hydrogel film to prepare miRNA@CaP NPs in situ on the patterned hydrogel film (3 mm in width, 13 mm in length and 1 mm in height). The grooves (0.5 mm in length) in the upper layer of the patterned hydrogel film for depositing KGN@PDA NPs, while the grooves (2.5 mm length) in the lower layer are for depositing miRNA@CaP NPs. The hydrogel patch was then rolled into a cylindrical

shape to create the directionally osteochondral plug (4 mm in diameter and 3 mm in height).

The free radical scavenging capability was confirmed by three different types of free radical, namely 1, 1-diphenyl-2-picrylhydrazyl (DPPH), 2, 2'-azino-bis (3-ethylbenzothiazoline-6-sulfonic acid) (ABTS), and 2-phenyl-4, 4, 5, 5-tetramethylimidazoline-1-oxyl 3-oxide (PTIO•) [S4, S5].

The drug release behavior was assessed by placing the prepared hydrogels in 20 mL of buffer solution at pH 6.0 (simulating the environment of an injured joint) and gently agitating them in a thermostatic rotary shaker at 100 rpm and 37°C. The concentrations of KGN and miR-26a were determined using high-performance liquid chromatography (LC-20; Shimadzu, Kyoto, Japan) and UV-vis spectroscopy, respectively.

S1.3 Cell Viability/Proliferation/Migration Assay and Cytotoxicity of Hydrogels Assay

Bone marrow-derived MSCs and chondrocytes were harvested using previously established methods [6]. Cell viability and proliferation were characterized by the Cell Counting Kit-8 (CCK-8) assay, while cell migration was recorded via a scratch assay. The hydrogels were subcutaneously implanted in rabbits to assess degradation and inflammation response. Blood biochemistry (Cr, ALT, AST, BUN, LDH, CK) was analyzed at 7 and 14 days post-implantation.

The effects of GTU-Fe/KGN@PDA and GTU-Fe/miRNA@CaP on chondrogenic and osteogenic differentiation of MSCs, respectively, were evaluated using a co-culture system. A co-culture system involves adding nanoparticles to a cell culture medium and incubating them for a certain period of time. The molecules in the nanoparticles will release continuously and act on the cells.

For chondrogenic differentiation, when we explored KGN@PDA inducing chondrogenic differentiation of MSCs, 5×10^5 cells were added to a 15mL centrifuge tube, and 1mL culture medium (KGN = 100 nM) was added. The control group did not contain KGN@PDA. After incubation for 48 hours, cartilage pellets could be found, which could be suspended by shaking. The old culture medium was removed, and 1mL complete culture medium was added to each tube. Afterwards, it was replaced every 3 days. After 14 days, 4% PFA fixed, OCT embedded, frozen sectioned, stained with Alcian Blue, and observed under the microscope.

For osteogenic differentiation of MSCs induced by miRNA@CaP in vitro, 5×10^4 cells/well were added into a six well plate, incubated for 24 hours, and replaced with the complete culture medium containing miRNA@CaP (miR-26a = 50 nM), and the control group did not contain miRNA@CaP. The culture medium was replaced every 3 days. After 7 days later, 4% PFA fixed, calcium nodules were observed by Alizarin red staining.

To further validate at the protein molecular level, we conducted Western-blot test separately. In short, 5×10^4 cells/well were added to a six well plate, incubated for 24 hours, and the old culture medium was replaced by the complete culture medium, which contained KGN@PDA or miRNA@CaP, the control group did not contain any nanoparticle. They were replaced every 3 days and cultured for 14 and 7 days, respectively. Cells were lysed and proteins were extracted. In the process of verifying the biological mechanism, it is necessary to shorten the culture time and extract the protein after adding complete culture medium for 30 minutes, 60 minutes, and 120 minutes, respectively.

Western blot analysis was performed according to standard procedures. The cells were lysed with RIPA buffer per the manufacturer's instructions, and supernatants were collected. Target bands were developed using an enhanced chemiluminescence kit (Millipore, Billerica, MA, USA) and analyzed with Quantity One software (ver. 4.6.6; Bio-Rad, Hercules, CA, USA). Primary antibodies detected Col II, Sox9, aggrecan, COL I, ALP, RUNX2, GSK-3β, p-β catenin, p-RUNX1, and p-c-Jun NH2-terminal protein kinase (p-JNK). GAPDH was used as the control.

The cellular uptake of miRNA@CaP by MSCs was visualized by confocal laser scanning microscopy (CLSM) with Cy5-conjugated miR-26a. In terms of genomics, total RNA was isolated from MSCs treated with KGN or miR-26a using TRIzol reagent and purified with a mirVana miRNA Isolation Kit. The R package Cluster Profiler was used for Kyoto Encyclopedia of Genes and Genomes (KEGG), Gene Ontology (GO), and Reactome enrichment analyses, as well as data visualization.

S1.4 In Vivo Osteochondral Defects Repair and Assessment

Forty female New Zealand White rabbits, weighing between 2.5 to 3.0 kg and aged 4-6 months, were utilized for this study. All animal procedures were performed in accordance with guidelines approved by the Ethics Committee of Zhejiang University (Approval NO.ZJCLA-IACUC-20010092). A cylindrical osteochondral defect, 4 mm in diameter and 3 mm deep, was created in the patellar groove using a stainless-steel punch. The osteochondral defects were either left untreated (control, group I) or filled with GTU-Fe (group II), GTU-Fe/KGN@PDA (group III), GTU-Fe/miRNA@CaP (group IV), or GTU-Fe/KGN@PDA/miRNA@CaP (group V), with 16 defects per group. The joint capsule and skin were sutured using interrupted stitches.

At 6 and 12 weeks, three defects were collected from each group and processed for gene expression analysis by qRT-PCR. The primer sequences used are listed in Table S2.

Samples from each group were independently examined and photographed by three individuals who were blind to the group allocations. The repair quality was evaluated using the criteria of the International Cartilage Repair Society (ICRS) Macroscopic Evaluation of Cartilage Repair (Table S3).

The covalent bond of Cy5 fluorescent dye was linked with miR-26a, synthesizing miR-26a-Cy5, by employing fluorescence labeling technology. The osteochondral defects were then examined using a small animal in vivo fluorescence imaging system (Maestro; CRi Inc., Woburn, MA, USA). For observation under confocal laser scanning microscopy (CLSM; LSM 510; Zeiss), the samples were fixed in neutral buffered formalin, decalcified, and placed into a 0.1 mol L^{-1} phosphate buffer solution containing 30% sucrose for 48 hours at 4°C, before being embedded into an optimal cutting temperature (OCT) compound. Coronal plane sections (10 μm) were cut using a cryostat (Leica, Wetzlar, Germany), and images were captured after staining the sections with DAPI and Alexa Fluor® 488 phalloidin.

Osteochondral explants were examined using a SkyScan 1276 apparatus (Bruker, Kontich, Belgium; 85 kV, 200 μA) using an image pixel size of 10 μm. Image reconstruction and analysis were conducted using NRecon and CTAn software (Bruker), respectively. Trabecular parameters such as separation (Tb.Sp; μm) and thickness (Tb.Th; μm) were identified. Bone volume (BV) and the BV/total volume (TV) ratio were calculated for each sample, with six samples per group analyzed.

Subsequently, samples were fixed in 4% (v/v) paraformaldehyde, decalcified in 10% (w/v) EDTA, embedded in paraffin, and sectioned at a thickness of 5 µm. The sections were stained with hematoxylin and eosin (H&E) for morphological evaluations, and Safranin-O and Masson's trichrome for glycosaminoglycan (GAG) and collagen distribution analysis, respectively. Histological sections were selected at random and examined histomorphometrically under a light microscope using Image-Pro Plus software (Media Cybernetics Inc, Rockville, MD, USA). For an overall assessment, repaired tissues were graded by three blinded observers utilizing the ICRS Visual Histological Assessment Scale (Table S4).

The birefringence, orientation, and anisotropy of collagen networks within repaired tissues were evaluated using polarized light microscopy. The predominant angle of collagen fiber orientation in two separate regions, the superficial zone (1/3 total thickness) and the base zone (2/3 total thickness), was investigated using the directionality plugin in Image J (NIH, Bethesda, MD, USA), in accordance with the structure of native cartilage (i.e., superficial collagen aligned parallel to the cartilage plane, while collagen in the base zone grew perpendicularly into the subchondral bone).

For the evaluation of Col II expression, immunohistochemistry observation was performed. The content of Col II was compared among the five groups.

S1.5 Statistical Analysis

All numerical data are presented as the mean value ± standard deviation (SD), where 'n' denotes the number of samples analyzed. Statistical analysis was conducted using one-way analysis of variance (ANOVA), followed by the Tukey HSD post hoc test for all pairwise comparisons. All statistical analyses were carried out using SPSS 20.0 software (SPSS Inc., Chicago, IL, USA). For all experiments, p-values < 0.05 (indicated by *) and 0.01 (indicated by **) were considered statistically significant.

S2 Supplementary Figures and Tables

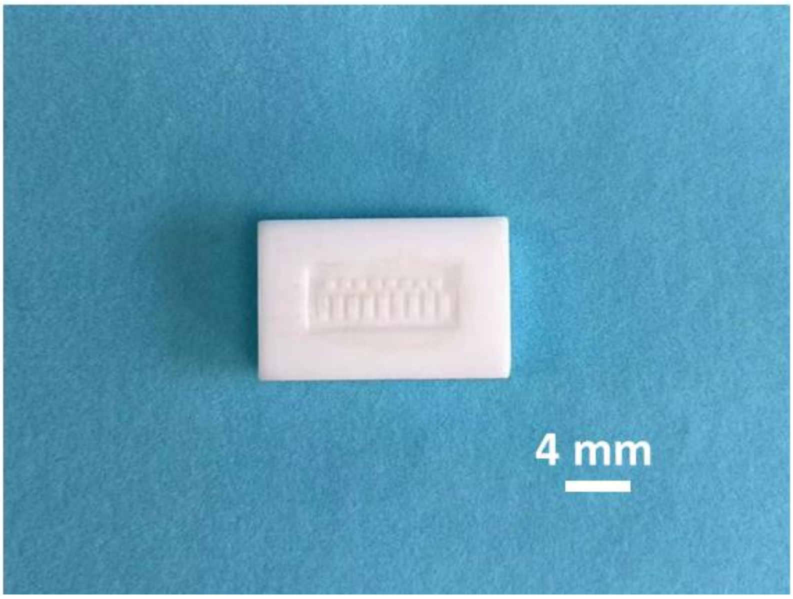

Fig. S1 The picture of PTFE mold

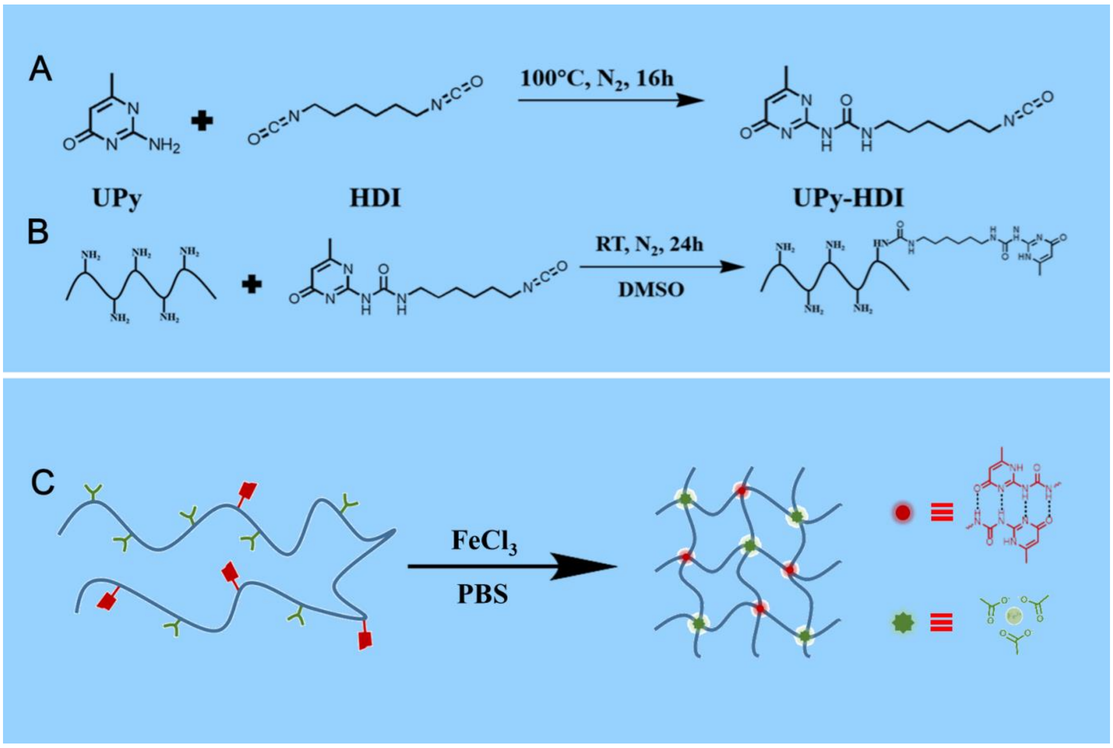

Fig. S2 (**A**) Synthesis of UPy-NCO. (**B**) Synthesis of UPy-NCO modified gelatin (GTU). (**C**) Fabrication of GTU-Fe hydrogel and the schematic diagram of the dual dynamic crosslinking in the hydrogel network

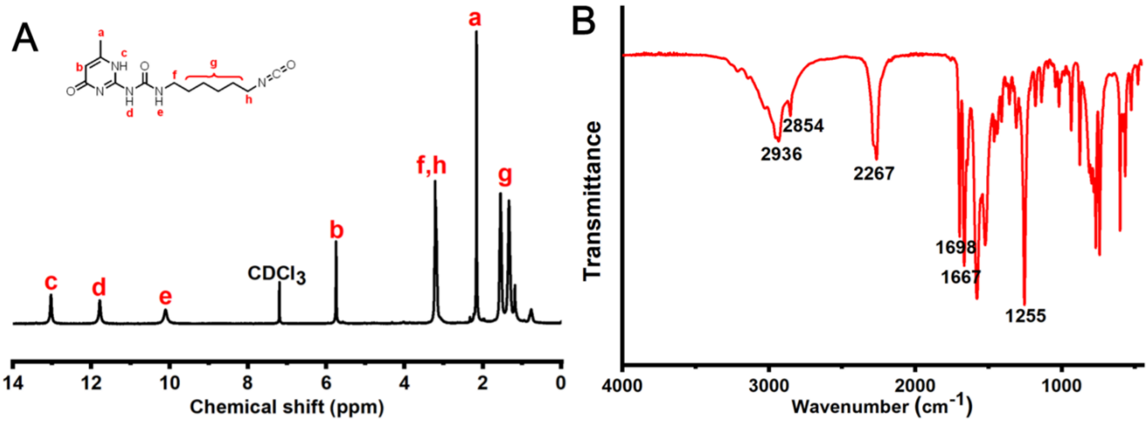

Fig. S3 (**A**) ^{1}H NMR and (**B**) FTIR absorption of UPy-NCO and UPy-NCO

UPy-NCO, which can form UPy-UPy dimers by quadruple hydrogen bonding in our system, was synthesized according to a previously reported method. ^{1}H NMR and Fourier transform infrared (FTIR) spectroscopy verified the synthesis of UPy-NCO successfully, and characteristic peaks are as followed.

^{1}H NMR (400 Hz, $CDCl_3$) (Fig. S3A): 13.03 ppm (-N**H**-C(-NH-)=N-), 11.79 ppm (-NH-C(-N**H**-)=N-), 10.11 ppm (-N**H**-CO-NH-CH_2-), 5.75 ppm (-CO-C**H**=C(CH_3)-), 3.22 ppm (-NH-C$\mathbf{H_2}$-(CH_2)$_4$-C$\mathbf{H_2}$-NCO), 2.16 ppm (-CO-CH=C(C$\mathbf{H_3}$)-) and 1.55-1.18 ppm (-NH-CH_2-(C$\mathbf{H_2}$)$_4$-CH_2-NCO).

FTIR (Fig. S3B): 2936 cm^{-1} and 2854 cm^{-1} (C-H symmetric stretching vibration of -CH_3 and -CH_2), 2267 cm^{-1} (-NCO), 1698 cm^{-1} and 1667 cm^{-1} (-C=O) and 1255 cm^{-1} (-C-N).

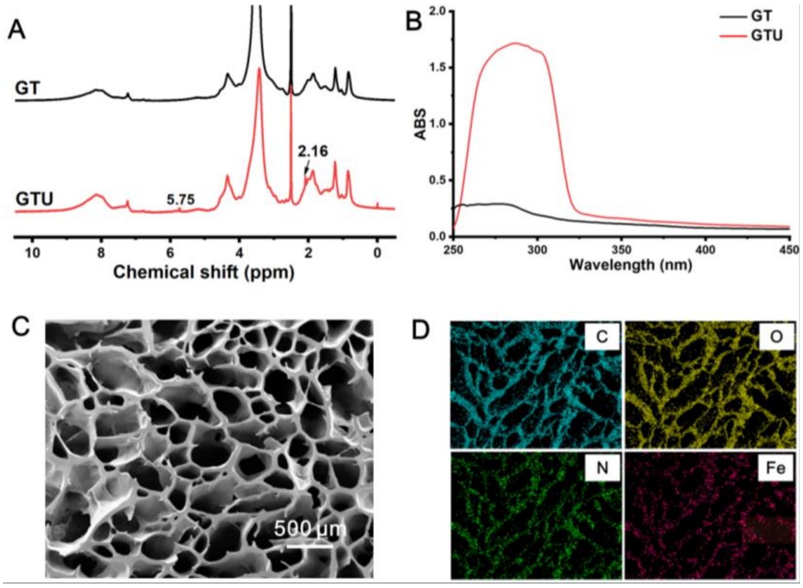

Fig. S4 (**A**) ^{1}HNMR and (**B**) UV absorption of GTU. (**C**) SEM and (**D**) EDS spectrum of GTU-Fe hydrogel

UPy group was introduced on the side chain of the gelatin by the reaction of NH_2 and NCO. ^{1}H NMR and UV-visible spectroscopy (Fig. S4B) carried out for gelatin-UPy and pure gelatin, which verified that the UPy was successfully grafted on the side chain of the gelatin.

^{1}H NMR (Fig. S4A): 5.75 ppm (-CO-C<u>**H**</u>=C(CH_3)-) and 2.16 ppm (-CO-CH=C(C<u>**H_3**</u>)-), respectively.

The SEM images clearly demonstrate that GTU-Fe exhibit a porous structure (Fig. S4C). The C, O, N and Fe element are evenly distributed in the network of GTU-Fe, as shown in Fig. S4D.

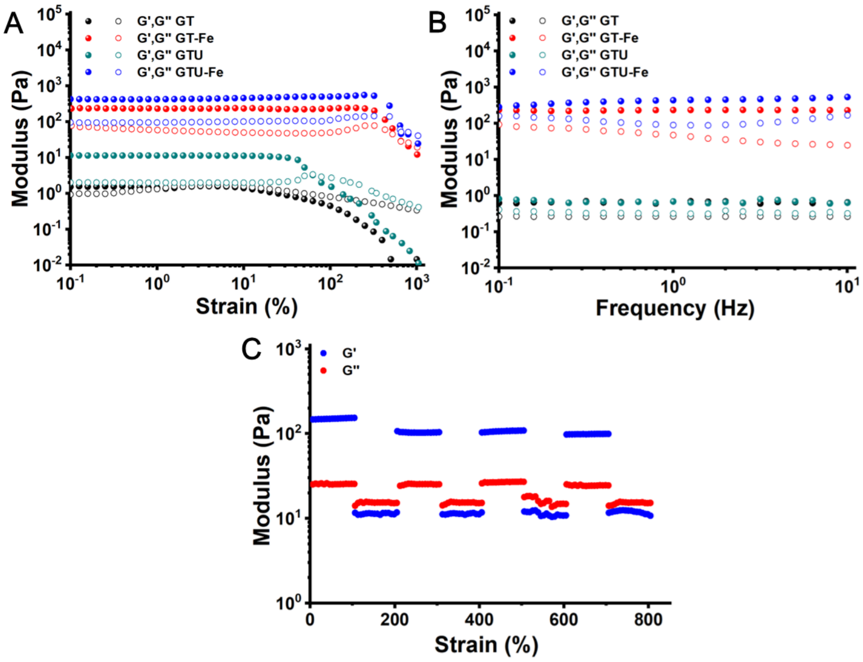

Fig. S5 (**A**) G' and G'' of the hydrogels with function of strain (0.1-1000%). (**B**) G' and G'' of the hydrogels with angular frequency (0.1-10 Hz). (**C**) Step-strain test at angular frequency 1 Hz (1% or 1000% of strain was applied)

The storage modulus (G') and loss modulus (G'') of GTU-Fe hydrogel were tested in order to evaluate mechanical features and the self-healing behavior further. Strain amplitude sweep were performed to analyze the elastic response of the hydrogels (Fig. S5A). For GTU-Fe hydrogel, no significant changes in G' and G'' were observed under low strains. While G' value decreased dramatically from 527 Pa to 77 Pa above the critical strain region (γ=321%). G' and G'' curves intersected at the strain 658%. With further increasing the strain, G' and G'' intensely decreased due to the collapse of the network. GTU-Fe hydrogel was in the state between solid and fluid near this critical point. Fig. S5B showed that G' (from 280 Pa to 529 Pa) and G'' (from 159 Pa to 166 Pa) increased with the increase of frequency, and G'＞G'' all the time, which indicated that the hydrogel was in a gelatinous state. Based on the strain amplitude sweep results, the step-strain test of GTU-Fe hydrogel was conducted to analyze the self-healing behavior (Fig. S5C). Initially, the hydrogel was treated under very low strain (γ=1%) without any obvious changes of G' and G''. But when the strain increased to 1000% and maintained for 100 s, G' value of GTU-Fe hydrogel decreased from 154 Pa to 12 Pa which was lower than G''. It indicated that the hydrogel network was disrupted. After the strain returned to 1%, G' recovered quickly to the 107 Pa and GTU-Fe hydrogel network was restored to the original state immediately. Even after four cycles, there was no obvious change observed. These results indicated that the network structure of GTU-Fe hydrogel could recover quickly after disruption, and the self-healing performance of GTU-Fe hydrogel was confirmed again.

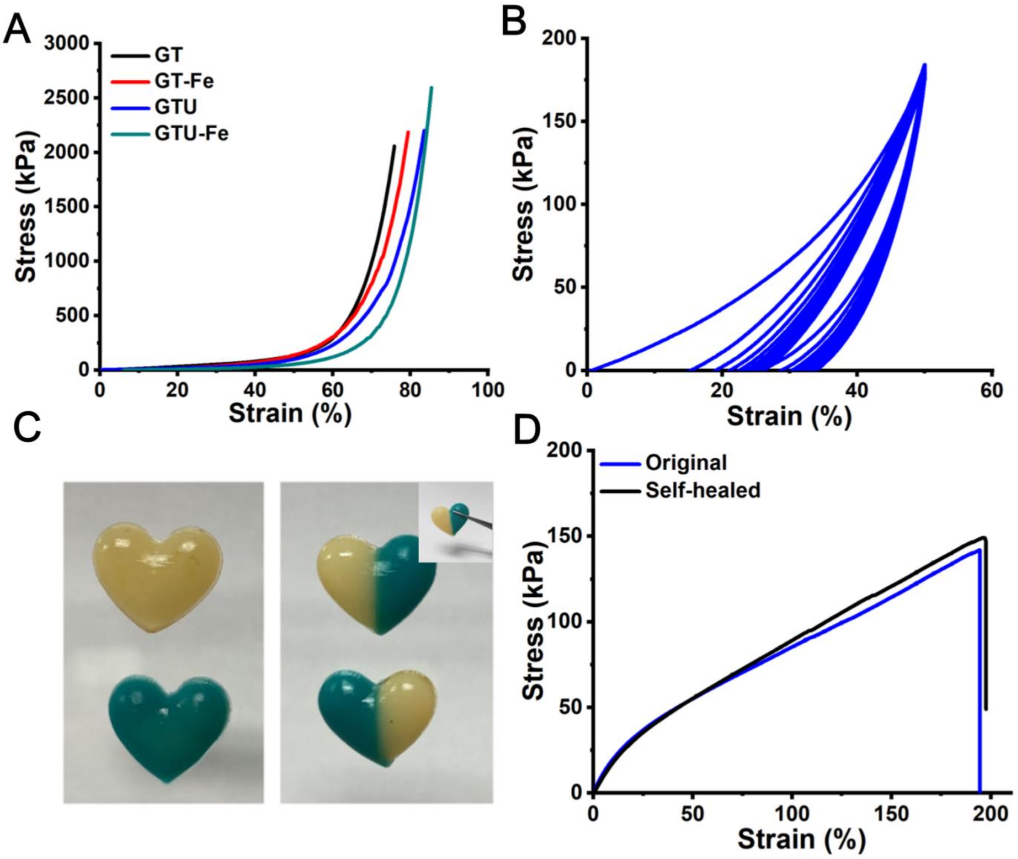

Fig. S6 (**A**) Compressive stress-strain curves of the hydrogels. (**B**) Cyclic compressive tests for GTU-Fe hydrogel with a constant stain of 50%. (**C**) Photograph of GTU-Fe hydrogel self-healed. (**D**) Tensile stress-strain curves of the hydrogels

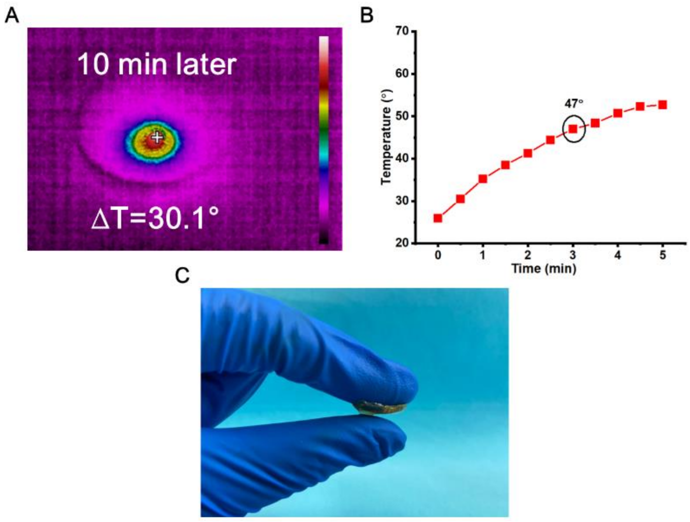

Fig. S7 (**A**) Infrared thermal images of the GTU-Fe hydrogel under NIR irradiation (808 nm, 1 W cm^{-2}, 10 cm). (**B**) Temperature enhancement of GTU-Fe hydrogel under irradiation with a NIR laser. (**C**) Adhesion behavior of GTU-Fe hydrogel after 3min irradiation

As shown in Fig. S7, the resulted GTU-Fe hydrogel can absorb and convert NIR light to heat. The temperature increments of GTU-Fe hydrogel were 54.8°C after irradiation for 10 min (808 nm, 1 W cm^{-2}, 10 cm) (Fig. S7A). The mild and controllable photothermal effect of the GTU-Fe hydrogel was shown in Fig. S7B. After an exposure of 3-min NIR , the temperature of GTU-Fe hydrogel has increased from 25.9°C to 47°C, accompanying with an excellent adhesion property and NIR-assisted roll-film capability (Fig. S7C).

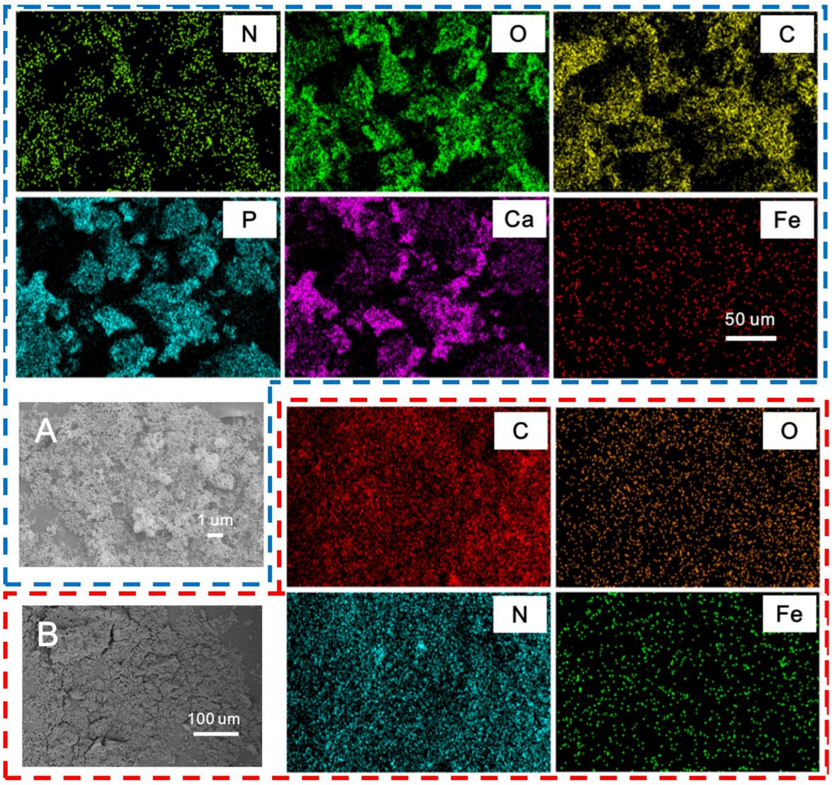

Fig. S8 (**A**) EDS spectrum of GTU-Fe/miRNA@CaP. (**B**) EDS spectrum of GTU-Fe/KGN@PDA

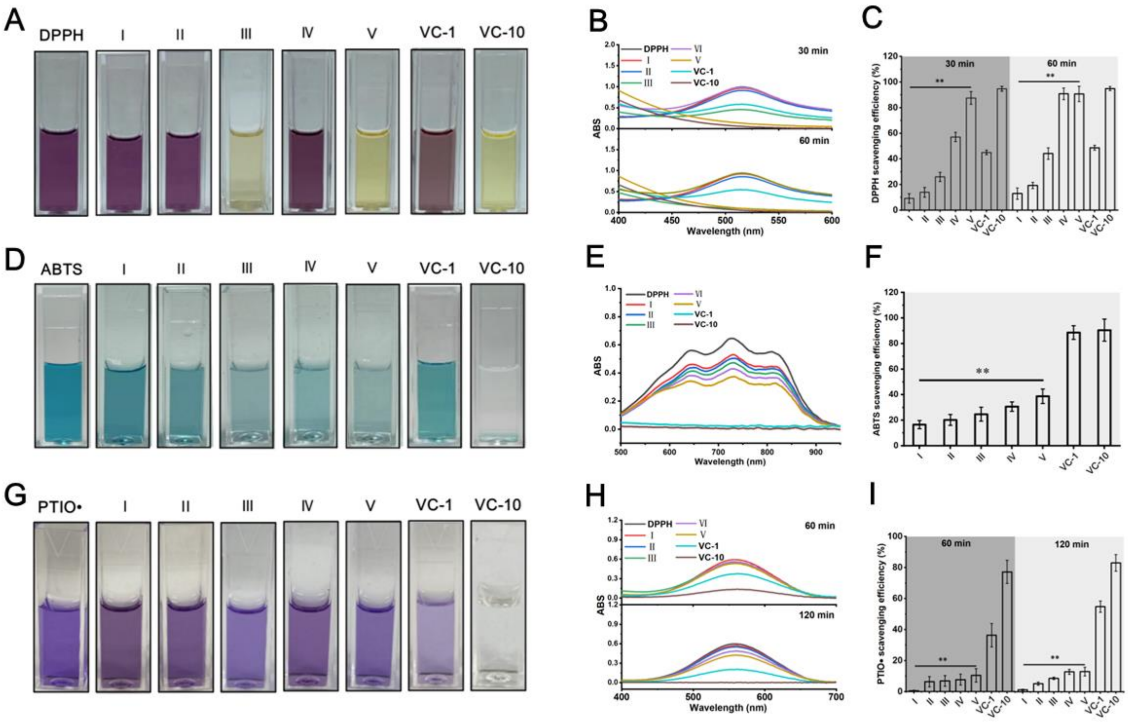

Fig. S9 Photograph of DPPH (**A**), ABTS (**D**) and PTIO• (**G**) solutions after reaction with hydrogel scaffold. UV-vis spectroscopy of DPPH (**B**), ABTS (**E**) and PTIO• (**H**) solution after reaction with hydrogel scaffold. DPPH (**C**), ABTS (**F**) and PTIO• (**I**) scavenging efficiency of hydrogel scaffold with different time point. (I: GT, II: GTU-Fe, III: GTU-Fe/KGN@PDA, IV: GTU-Fe/miRNA@CaP, V: GTU-Fe/KGN@PDA/miRNA@CaP, VC-1: 1 mM vitamin C/methanol solution, VC-10: 10 mM vitamin C/methanol solution)

As illustrated in Fig. S9A, the color of DPPH and hydrogel plug mixed solutions faded as the KGN@PDA NPs added and turned to yellow. The introduction of PDA enhanced the DPPH capture efficiency of hydrogel scaffold. The DPPH scavenging efficiency of hydrogel scaffold is analyzed from the absorption spectra as shown in Fig. S9B. According to the change of the characteristic absorption peak at 517 nm, it can be seen that the absorbance gradually decreases with the extension of time, indicating free radicals in the solution reduces. It can be seen from Fig. S9C that after the introduction of KGN@PDA, GTU-Fe/KGN@PDA can capture 57% within 30 min and 91% within 60 min. GTU-Fe/KGN@PDA/miRNA@CaP hydrogel scaffold can capture 88% within 30 min and 91% within 60 min, which was much higher than GT (9% within 30 min and 13% within 60 min). This phenomenon indicated that hydrogel plug possessed excellent ability to capture free radicals, and which was verified by ABTS method as well. The color of ABTS and hydrogel plug mixed solutions was observed to fade. We can draw the conclusion that GTU-Fe/KGN@PDA/miRNA@CaP hydrogel plug can capture 39% within 6 min from the data changes in the absorption peak at 734 nm. As shown in Fig. S9G, the scaffold can also trap PTIO• free radicals, and over time, the color of PTIO• and hydrogel scaffold mixed solutions fades. From the change of absorption peak at 557 nm, we can conclude that the free radical scavenging efficiency of the scaffold is 16%.

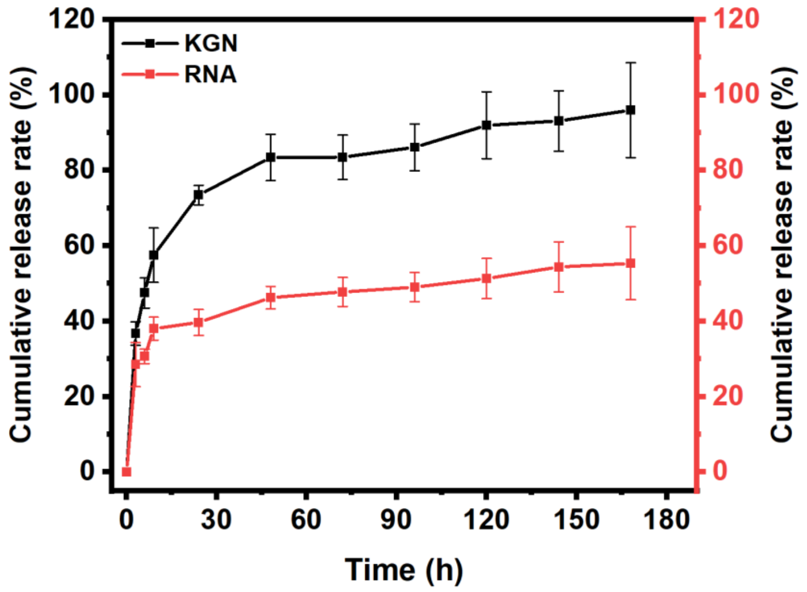

Fig. S10 Cumulative release rate of KGN and miR-26a in the simulated bone defect medium

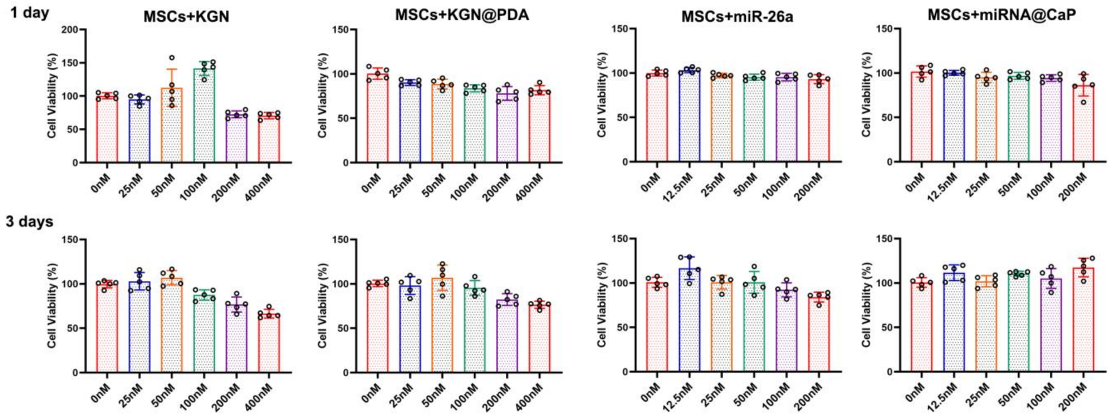

Fig. S11 Cell counting kit-8 assay was used to assess the proliferative effect of KGN and miR-26a, cytotoxicity and cytocompatibility of KGN@PDA and miRNA@CaP on MSCs at 1, 3 days. All data represent mean ± SD (n = 5)

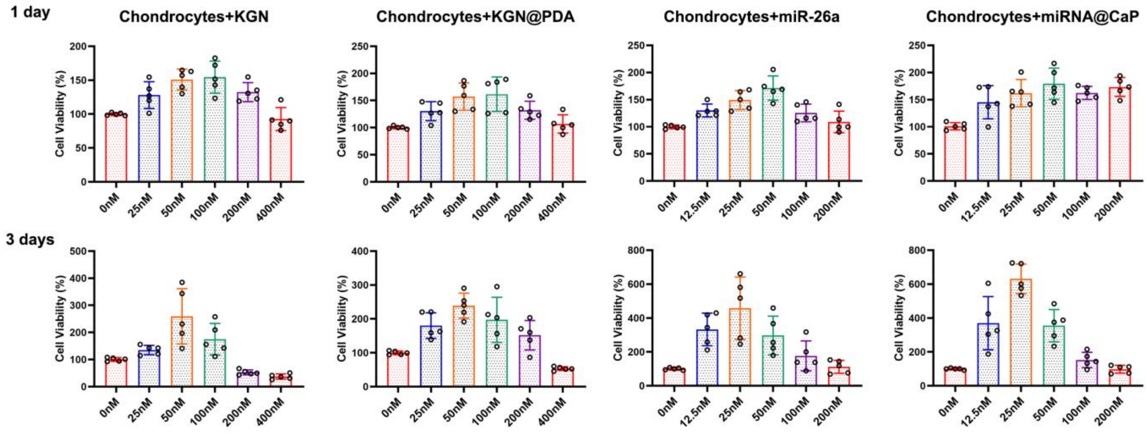

Fig. S12 Cell counting kit-8 assay was used to assess the proliferative effect of KGN and miR-26a, cytotoxicity and cytocompatibility of KGN@PDA and miRNA@CaP on chondrocytes at 1, 3 days. All data represent mean ± SD (n = 5)

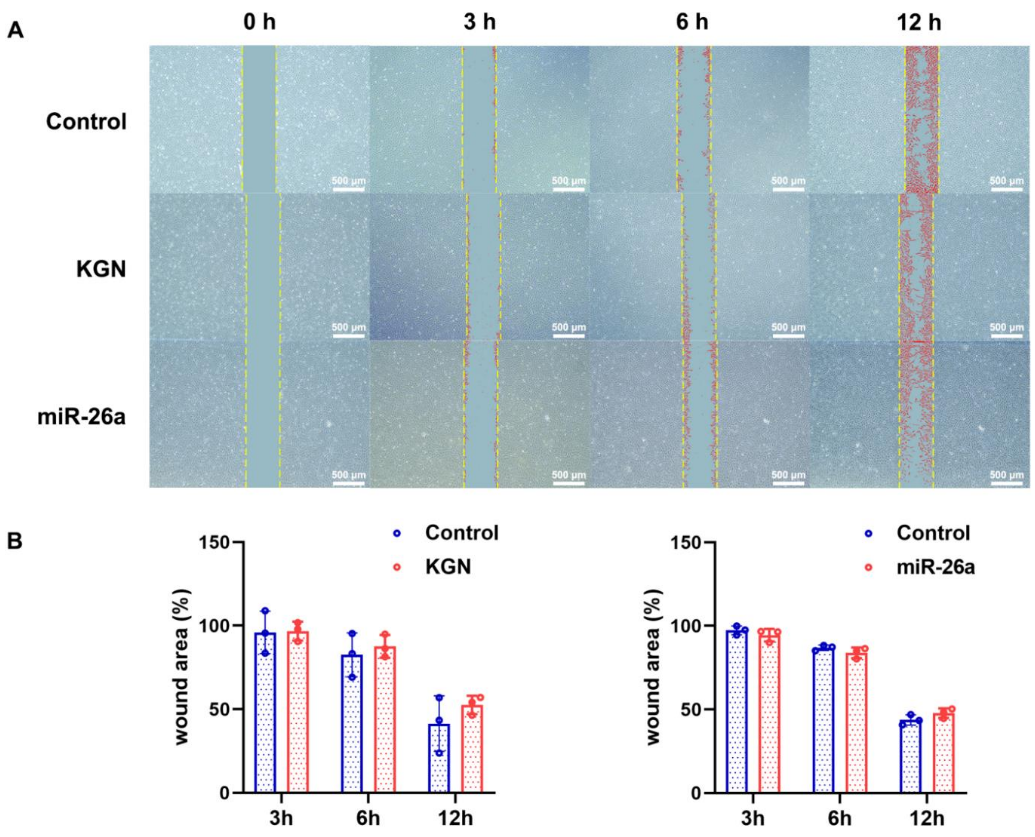

Fig. S13 (**A**) Evaluation of cell migration of MSCs with KGN (100 nM) or miR-26a (50 nM) for 0, 3, 6 and 12 h. Scale bar = 500 µm. (**B**) Quantification of wound area (%) of KGN or miR-26a group, relative to control, at different time points. All data represent mean ± SD (n = 3)

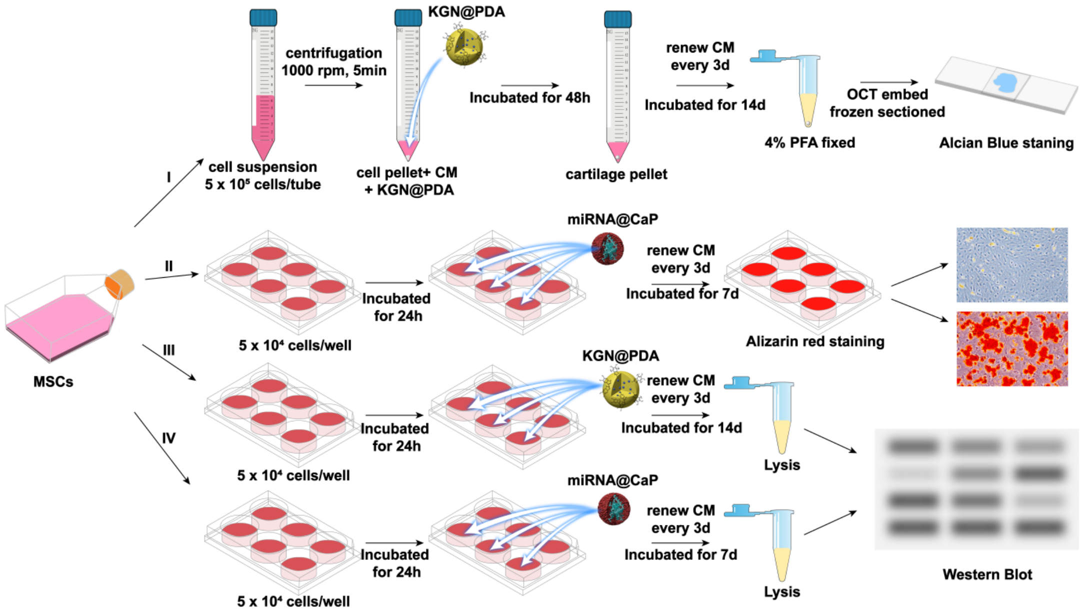

Fig. S14 Schematic illustrating of the co-culture system for assessing the impacts of GTU-Fe/KGN@PDA and GTU-Fe/miRNA@CaP on the chondrogenic and osteogenic differentiation of MSCs. I: cartilage microsphere culture; II: osteogenic differentiation; III: chondrogenic differentiation (Western Blot); IV: osteogenic differentiation (Western Blot)

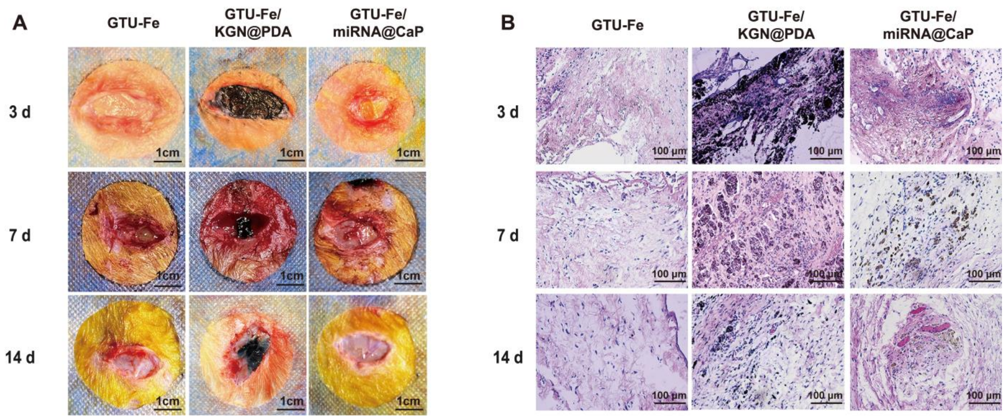

Fig. S15 (**A**) Gross morphology and (**B**) histological photographs of different materials (from left to right: GTU-Fe, GTU-Fe/KGN@PDA, GTU-Fe/miRNA@CaP) after implanted subcutaneously for 3, 7, 14 days. (A) Scale bar = 1 cm (B) Scale bar = 100 μm

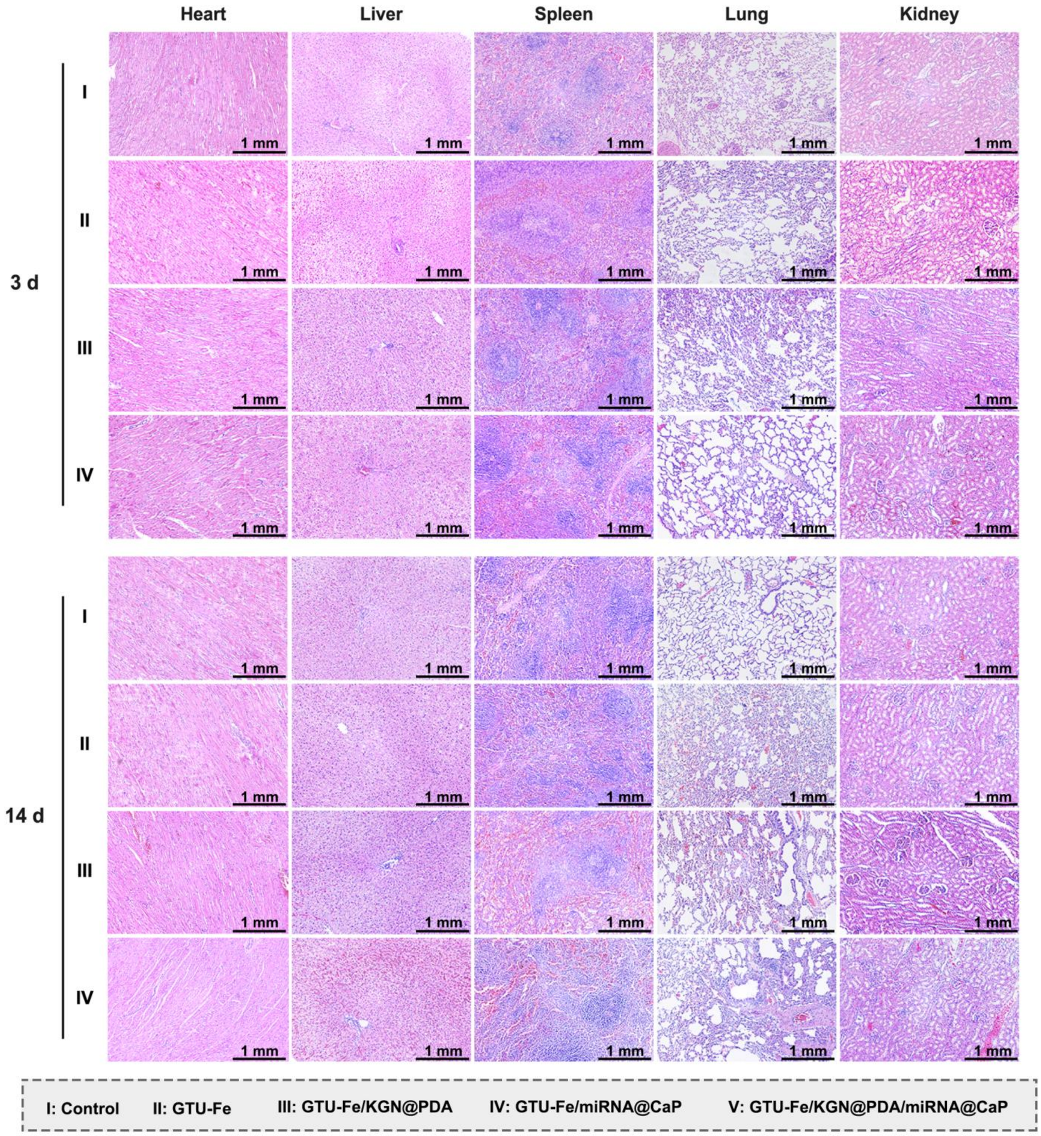

Fig. S16 Representative images of H&E staining of the visceral organs (from left to right: heart, liver, spleen, lung, kidney) in different groups at 3 & 14 days post-implantation. Scale bar = 1 mm

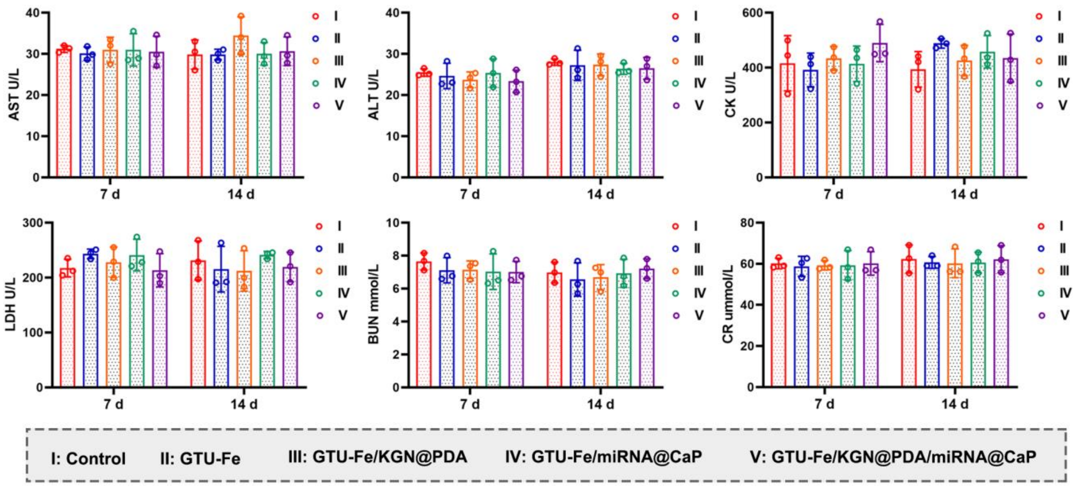

Fig. S17 Statistical chart of AST, ALT, CK, LDH, BUN and CR levels of different groups at 7 & 14 days after implanting. All data represent mean ± SD (n = 3)

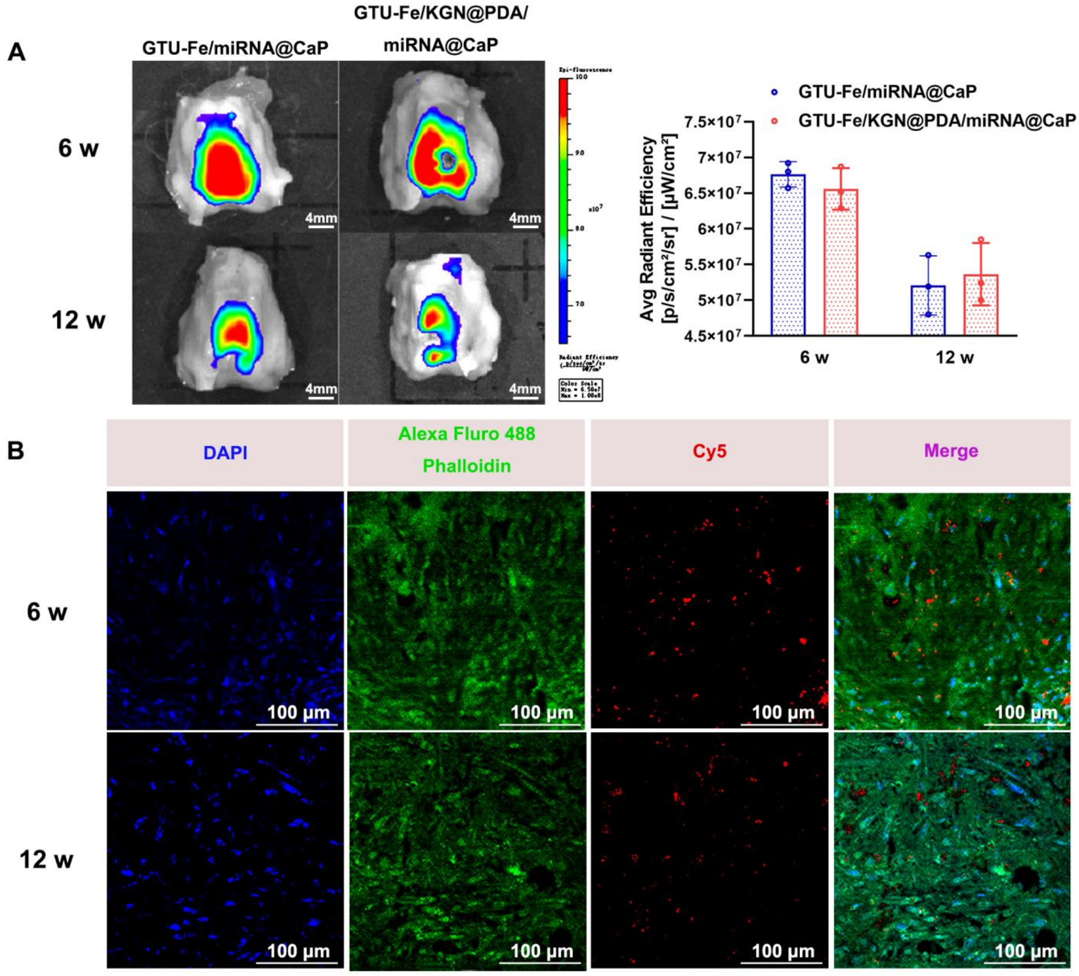

Fig. S18 Spatiotemporal validation of the therapeutic effect of miRNA@CaP in vivo. (**A**) The IVIS images of rabbits' distal femurs after implantion of the GTU-Fe/miRNA@CaP and GTU-Fe/KGN@PDA/miRNA@CaP, and statistics of average fluorescence intensity at 6&12 weeks. Scale bar = 4 mm. (**B**) Representative frozen section images showing distribution of miRNA@CaP in the zone of subchondral bone. Scale bar = 100µm. The p-value was calculated by one-way ANOVA test (*p < 0.05 and **p < 0.01). Data are presented as mean ± SD (n = 3)

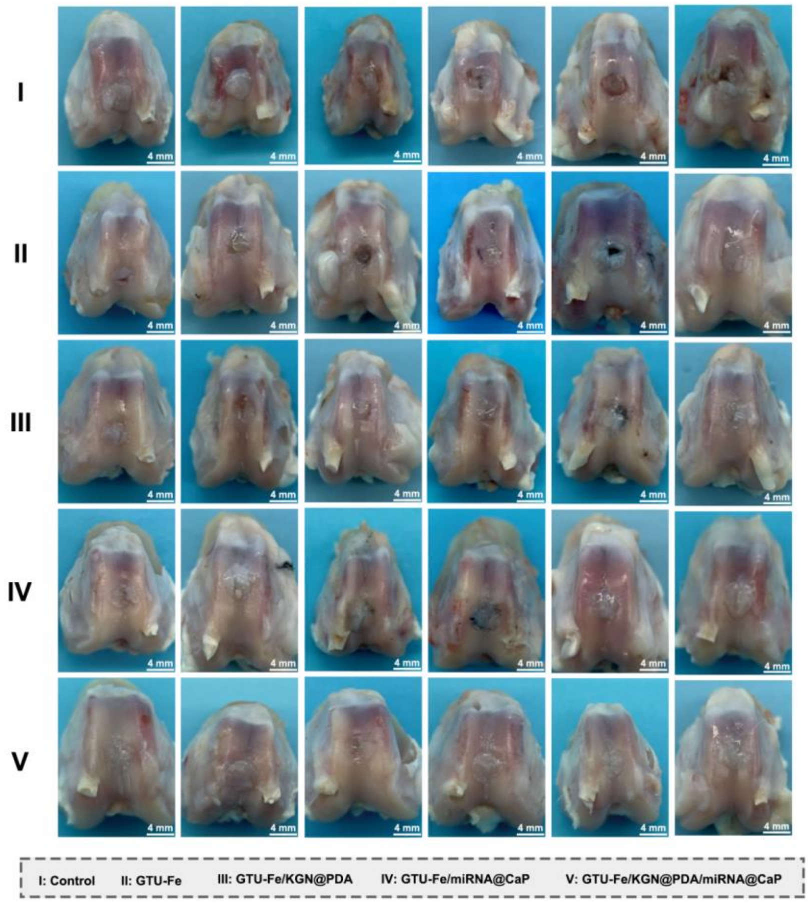

Fig. S19 All samples in each group at 12 weeks. Scale bar = 4mm

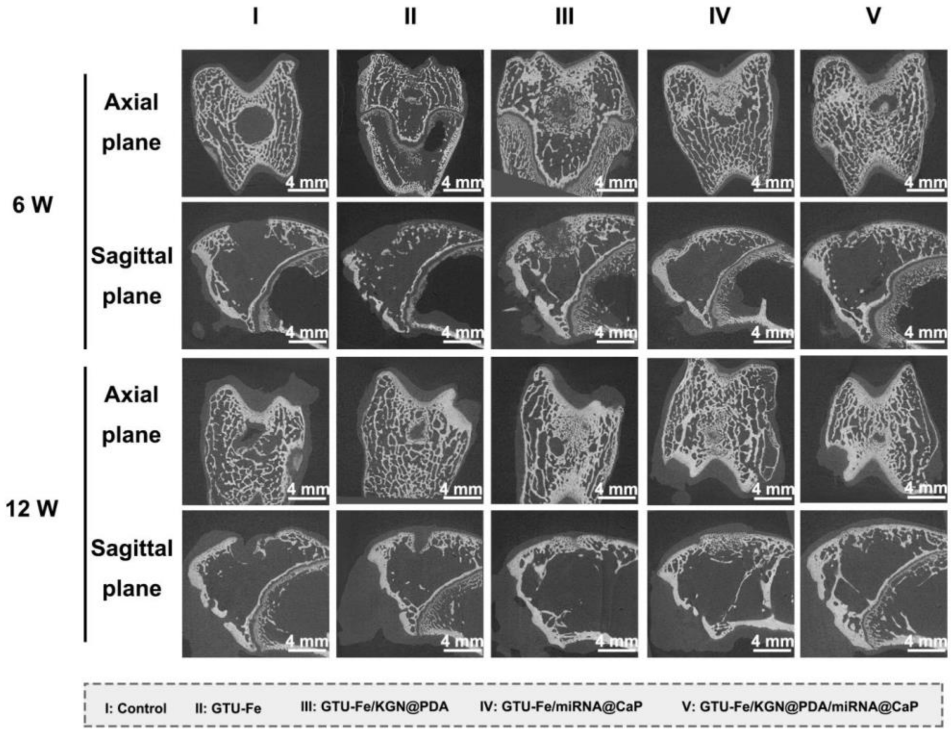

Fig. S20 Representative micro-CT images, including axial plane and sagittal plane, of subchondral bone in osteochondral defects at 6 & 12 weeks after implantation. Scale bar = 4 mm

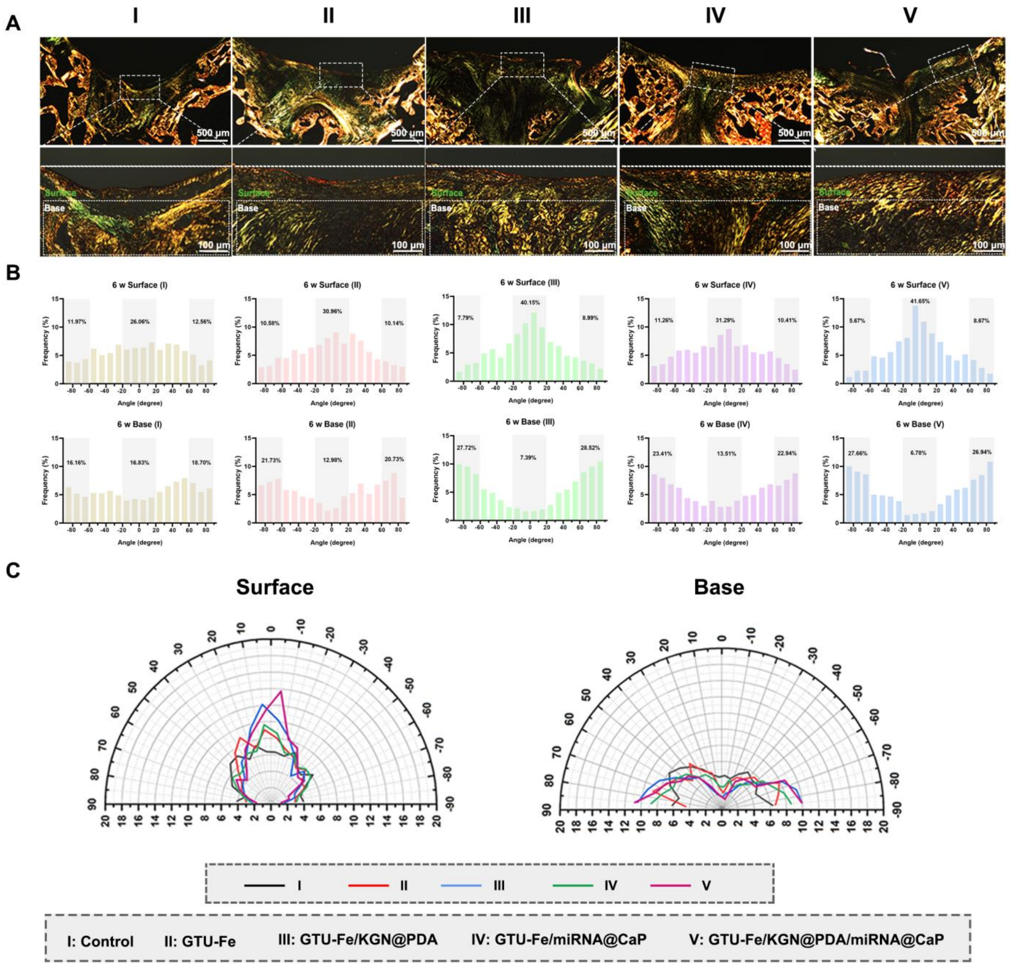

Fig. S21 Representative polarized light images and quantification of collagen distribution in cartilage and subchondral bone layer at 6 weeks. (**A**) Polarized light images of regenerated tissues after picrosirius red staining. Scale bar = 500 μm (upper); 100 μm (lower). (**B**) Quantitative analyses of the collagen fiber distribution in the surface area (upper 1/3) and base area (bottom 2/3). (**C**) Comprehensive comparison of collagen orientation within surface and base areas between the control, GTU-Fe, GTU-Fe/KGN@PDA, GTU-Fe/miRNA@CaP, and GTU-Fe/KGN@PDA/miRNA@CaP groups presented by polar coordinates

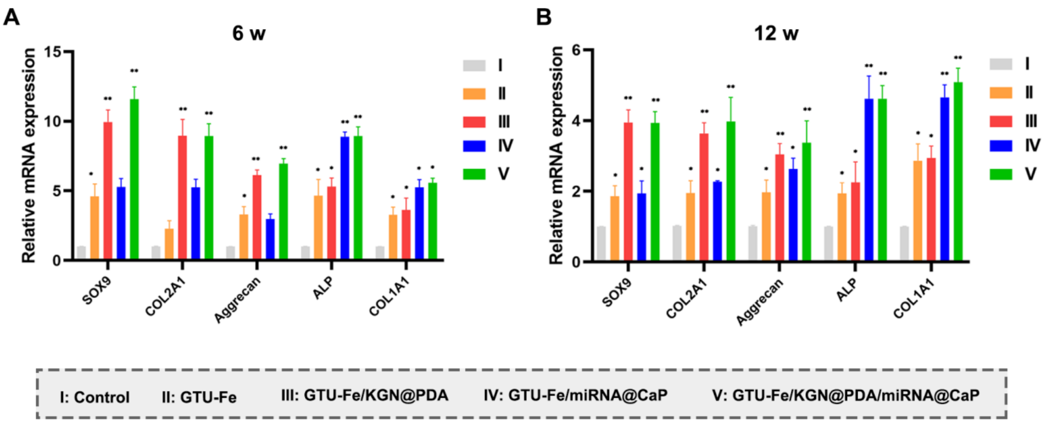

Fig. S22 qRT-PCR results of SOX9, COL2A1, Aggrecan, ALP, COL1A1 in different groups at 6 weeks (A) and 12 weeks (B). The p-value was calculated by one-way ANOVA test (*p < 0.05 and **p < 0.01). Data are presented as mean ± SD (n = 3)

Table S1 Relative of the primary antibody utilised in study

Antibody	Company	Experiment	Source
COL2A1	Affinity	In vivo/In vitro	Rabbit
Aggrecan	Affinity	In vitro	Rabbit
SOX9	Affinity	In vitro	Rabbit
COL1A1	Proteintech	In vitro	Rabbit
ALP	Affinity	In vitro	Rabbit
RUNX2	Affinity	In vitro	Rabbit
P- RUNX1	Affinity	In vitro	Rabbit
GSK3β	Affinity	In vitro	Rabbit
P-beta Catenin	Affinity	In vitro	Rabbit
P-JNK	Affinity	In vitro	Rabbit
GAPDH	Beyotime	In vitro	Rabbit

Table S2 Nucleotide primers used for RT-PCR

Genes	Forward (5'-3’)	Reverse (5'-3)
Aggrecan	F:GTGGTGGTGAAAGGTGTTGTG	R:GGTGGAAGCCATCCTCGTAG
SOX9	F:CAGACTGTTGTCACCTGTCCCTC	R:GGTTGACCATACCCTCTGAAGAA
COL2A1	F:CAACAACCAGATCGAGAGCA	R:GCTCCACCAGTTCTTCTTGG
COL1A1	F:CTTGCTTGAAGACCCGAGTGG	R:CTTTGGTTGCTGGCAGGACA
ALP	F:GCTGCAAGGACATCGCTTATC	R:TCGACCTCATACTCCACGTCAG
GAPDH	F:AGAGCACCAGAGGAGGACGAG	R：CTGGGATGGAAACTGTGAAGAG

Table S3 International cartilage repair society macroscopic evaluation of cartilage repair

Cartilage repair assessment ICRS	Points
Degree of defect repair	
In level with surrounding cartilage	4
75% repair of defect depth	3
50% repair of defect depth	2
25% repair of defect depth	1
0% repair of defect depth	0
Integration to the border zone	
Complete integration with surrounding cartilage	4
Demarcating border < 1 mm	3
3/4th of graft integrated, 1/4th with a notable border > 1 mm width	2
1/2 of graft integrated with surrounding cartilage, 1/2 with a notable order > 1 mm	1
From no contact to 1/4th of graft integrated with surrounding cartilage	0
Macroscopic appearance	
Intact smooth surface	4
Fibrillated surface	3
Small, scattered fissures or cracks	2
Several small or few but large fissures	1
Total degeneration of the grafted area	0
Overall repair assessment	
Grade I: normal	12
Grade II: nearly normal	11-8
Grade III: abnormal	7-4
Grade IV: severely abnormal	3-0

Table S4 International cartilage repair society visual histological assessment scale for cartilage repair

Feature	Points
Surface	
Smooth/continuous	3
Discontinuities/irregularities	0
Matrix	
Hyaline	3
Mixture: hyaline/fibrocartilage	2
Fibrocartilage	1
Fibrous tissue	0
Cell distribution	
Columnar	3
Mixed/columnar clusters	2
Clusters	1
Individual cells/disorganized	0
Cell population viability	
Predominantly viable	3
Partially viable	1
<10% viable	0
Subchondral bone	
Normal	3
Increased remodeling	2
Bone necrosis/granulation tissue	1
Detached/fracture/callus at base	0
Cartilage mineralization (calcified cartilage)	
Normal	3
Abnormal/inappropriate location	0

Supplementary References

[S1] P.Y. Dankers, E.N. van Leeuwen, G.M. van Gemert, A.J. Spiering, M.C. Harmsen et al., Chemical and biological properties of supramolecular polymer systems based on oligocaprolactones. Biomaterials **27**(32), 5490-5501 (2006). https://doi.org/10.1016/j.biomaterials.2006.07.011

[S2] Y. Xu, H. Yang, H. Zhu, L. Jiang, H. Yang, Self-healing gelatin-based shape memory hydrogels via quadruple hydrogen bonding and coordination crosslinking for controlled delivery of 5-fluorouracil. J. Biomater. Sci. Polym. Ed. **31**(6), 712-728 (2020). https://doi.org/10.1080/09205063.2020.1713711

[S3] Y. Liang, Z. Li, Y. Huang, R. Yu, B. Guo, Dual-dynamic-bond cross-linked antibacterial adhesive hydrogel sealants with on-demand removability for post-wound-closure and infected wound healing. ACS Nano **15**(4), 7078-7093 (2021). https://doi.org/10.1021/acsnano.1c00204

[S4] X. Qi, X. Tong, S. You, R. Mao, E. Cai et al., Mild hyperthermia-assisted ros scavenging hydrogels achieve diabetic wound healing. ACS Macro. Lett. **11**(7), 861-867 (2022). https://doi.org/10.1021/acsmacrolett.2c00290

[S5] X. Li, 2-phenyl-4,4,5,5-tetramethylimidazoline-1-oxyl 3-oxide (ptio(*)) radical scavenging: A new and simple antioxidant assay in vitro. J. Agric. Food Chem. **65**(30), 6288-6297 (2017). https://doi.org/10.1021/acs.jafc.7b02247

[S6] Y. Qi, W. Zhang, G. Li, L. Niu, Y. Zhang et al., An oriented-collagen scaffold including wnt5a promotes osteochondral regeneration and cartilage interface integration in a rabbit model. FASEB J. **34**(8), 11115-11132 (2020). https://doi.org/10.1096/fj.202000280R
